# Class VI G protein-coupled receptors in *Aspergillus oryzae* regulate sclerotia formation through GTPase-activating activity

**DOI:** 10.1007/s00253-023-12862-0

**Published:** 2024-01-17

**Authors:** Dong Min Kim, Itsuki Sakamoto, Manabu Arioka

**Affiliations:** 1 Department of Biotechnology, The University of Tokyo, 1-1-1 Yayoi, Bunkyo-ku, Tokyo, 113-8657 Japan; 2 Collaborative Research Institute for Innovative Microbiology (CRIIM), The University of Tokyo, 1-1-1 Yayoi, Bunkyo-ku, Tokyo, 113-8657, Japan

**Keywords:** *Aspergillus oryzae*, Class VI GPCR, G proteins, Regulator of G protein signaling (RGS), Sclerotia, *velB*

## Abstract

**Abstract:**

G protein-coupled receptors (GPCRs) comprise the largest family of transmembrane receptors in eukaryotes that sense and transduce extracellular signals into cells. In *Aspergillus oryzae*, 16 canonical GPCR genes are identified and classified into nine classes based on the sequence similarity and proposed functions. Class VI GPCRs (AoGprK-1, AoGprK-2, and AoGprR in *A. oryzae*), unlike other GPCRs, feature a unique hybrid structure containing both the seven transmembrane (7-TM) and regulator of G-protein signaling (RGS) domains, which is not found in animal GPCRs. We report here that the mutants with double or triple deletion of class VI GPCR genes produced significantly increased number of sclerotia compared to the control strain when grown on agar plates. Interestingly, complementation analysis demonstrated that the expression of the RGS domain without the 7-TM domain is sufficient to restore the phenotype. In line with this, among the three Gα subunits in *A. oryzae*, AoGpaA, AoGpaB, and AoGanA, forced expression of GTPase-deficient mutants of either AoGpaA or AoGpaB caused an increase in the number of sclerotia formed, suggesting that RGS domains of class VI GPCRs are the negative regulators of these two GTPases. Finally, we measured the expression of velvet complex genes and sclerotia formation-related genes and found that the expression of *velB* was significantly increased in the multiple gene deletion mutants. Taken together, these results demonstrate that class VI GPCRs negatively regulate sclerotia formation through their GTPase-activating activity in the RGS domains.

**Key points:**

*• Class VI GPCRs in A. oryzae regulate sclerotia formation in A. oryzae*

*• RGS function of class VI GPCRs is responsible for regulation of sclerotia formation*

*• Loss of class VI GPCRs resulted in increased expression of sclerotia-related genes*

**Supplementary Information:**

The online version contains supplementary material available at 10.1007/s00253-023-12862-0.

## Introduction

G protein-coupled receptors (GPCRs) are the largest group of transmembrane (TM) receptors containing over 900 genes in humans. They share a common basic structure composed of seven transmembrane (7-TM) domains, ligand-binding extracellular N-terminal tail, and intracellular C-terminus that interacts with G proteins (Bockaert and Pin 1999). GPCR ligands include endogenous molecules such as neuropeptides, amino acids, biogenic amines, chemokines, lipid mediators, nucleotides, polypeptide hormones, and exogenous molecules including viruses, photons of light, and olfactory and gustatory molecules (Civelli et al. 2013). The classical signal transduction through GPCRs is dependent on receptor-mediated activation of heterotrimeric G proteins at the plasma membrane, which are composed of three subunits, Gα, Gβ, and Gγ. G proteins have apparently universal roles as signaling proteins in eukaryotes. The Gα subunit binds GTP and GDP and hydrolyzes GTP to GDP, and the Gβ and Gγ subunits form a heterodimer. In the inactive GDP-bound state, the three subunits are present in a complex in association with a GPCR. Ligand binding to the GPCR leads to an exchange of GDP to GTP on the Gα subunit and dissociation of Gα and Gβγ heterodimer. Both the Gα-GTP and Gβγ moieties regulate downstream effector proteins in various systems, including ion channels, adenylyl cyclases, phosphodiesterases, and phospholipases. GTP hydrolysis on the Gα subunit allows the GDP-bound Gα to re-associate with the Gβγ heterodimer and the GPCR at the membrane, ready to reinitiate the signaling cycle (McCudden et al. 2005).

Regulator of G protein signaling (RGS) is a family of multifunctional signal transduction proteins with highly diversified functions. RGS family members share a conserved core RGS domain of 130 amino acids which directly binds to the activated Gα subunit and negatively regulates G protein signaling by significantly stimulating its GTPase activity. Binding of RGS stabilizes Gα-GTP in its transition-state intermediate form and thus lowers the reaction free energy required for GTP hydrolysis. In mammals, more than 30 members of the RGS protein family have been reported (Chidiac and Roy 2003;Siderovski and Willard 2005;Yu 2006).

In *Aspergillus* spp., about 15 canonical GPCR genes have been identified in an individual organism, which are classified into nine classes (Supplemental Table S1; Affeldt et al. 2014). Functional studies of fungal GPCRs have been actively conducted, demonstrating that they play important roles in morphogenesis, reproduction, virulence, and secondary metabolite production (Lengeler et al. 2000;Yu and Keller 2005;Zhang et al. 2011). Among the fungal GPCRs, class VI GPCRs are unique in that they possess a hybrid structure containing N-terminal 7-TM and C-terminal RGS domains. This type of GPCRs is present in fungi and plants, but not in animals (Chen et al. 2003;Urano et al. 2013;Wang et al. 2013). AtRGS1 in *Arabidopsis thaliana* is the first GPCR with this hybrid structure to be reported (Chen et al. 2003). Unlike standard GPCRs, AtRGS1 interacts with self-activating Gα subunit AtGPA1 and triggers hydrolysis of bound GTP and inactivation, but not exchange of GDP-GTP (Johnston et al. 2007). In addition, it has been reported that AtRGS1 regulates plant cell proliferation and is involved in sugar signaling such as glucose, fructose, and sucrose and mediates jasmonate responses (Chen and Jones 2004;Li et al. 2019a). AtRGS1 also promotes the formation of autophagosomes during D-glucose stimulation (Yan et al. 2017). In filamentous fungi, class VI GPCRs GprK and GprR of *Aspergillus flavus* are commonly involved in carbon source sensing, conidia formation, and osmotic pressure- and pH stress-sensing (Affeldt et al. 2014). It has also been reported that GprK and GprR play roles in cell wall stress and oxylipin sensing, respectively. In *Aspergillus fumigatus*, GprK has been reported to be involved in carbon source sensing, oxidative stress response, regulation of secondary metabolism, virulence, and functions in downregulating the PKA-germination pathway (Jung et al. 2016). Furthermore, Morgs7 of the pathogenic fungus *Magnaporthe oryzae* that belongs to GprK family has been reported to be involved in cAMP signaling, hydrophobic surface sensing, and virulence (Zhang et al. 2011;Li et al. 2019b; Xu et al. 2023). Recently, MrGprK in *Metarhizium robertsii* was shown to contribute to vegetative growth, conidia production, pH regulation, appressorium formation, and cuticle penetration (Yu et al. 2021). Thus, the accumulating evidence implicates the class VI GPCRs in various important cellular functions in the filamentous fungi, although it is unknown whether they play roles as ligand-sensing canonical GPCRs or as RGSs.

In this study, we characterized the physiological roles of class VI GPCRs in *Aspergillus oryzae*. *A. oryzae* has been extensively studied with a primary focus on breeding techniques and development of methods for sake brewing, but recently, it has also gained attention as a cell biology research model, particularly in the fields of live cell imaging of organelles, protein vesicle movement, autophagy, and Woronin body function (Kitamoto 2015). Since two orthologues for GprK and one orthologue for GprR were found in the genome of *A. oryzae*, we generated three single, three double, and one triple gene deletion mutants and examined the phenotypes. We found that the double and triple gene deletion mutants, but not the single gene deletion mutants, produced significantly increased numbers of sclerotia when grown on agar plates. Interestingly, complementation experiments demonstrated that the C-terminal RGS domain, but not the N-terminal 7-TM domain, was responsible for the regulation of sclerotia formation. GTPase-activating activity (GAP activity) of the RGS domains of class VI GPCRs was confirmed in an experiment using the yeast pheromone pathway. In line with the suggested roles of class VI GPCRs as RGSs, forced expression of the active form of Gα subunits resulted in the increase in sclerotia formation. qRT-PCR analysis demonstrated that the multiple gene deletion mutants exhibited increased expression of velvet protein-encoding gene *velB*, and deletion of *velB* resulted in the decrease in the sclerotia formation in the triple gene deletion mutant. Taken together, these results demonstrate that class VI GPCRs in *A. oryzae* negatively regulate sclerotia formation by controlling the expression of development-related gene via their GAP activity.

## Materials and methods

### Strains and media

*A. oryzae* strains used in this study are listed in Supplemental Table S2. NSPlD1 (Maruyama and Kitamoto 2008), a derivative of RIB40 (National Research Institute of Brewing, Hiroshima, Japan), was used as the wild type strain and transformation host. Dextrin-peptone-yeast extract medium (DPY; 2% dextrin, 1% polypeptone, 0.5% yeast extract, 0.5% KH_2_PO_4_, and 0.05% MgSO_4_·7H_2_O (pH 5.5)) containing 0.1% each of uridine and uracil and Czapek-Dox (CD) medium (3 g/l NaNO_3_, 2 g/l KCl, 1 g/l KH_2_PO_4_, 0.5 g/l MgSO_4_·7H_2_O, 0.002 g/l FeSO_4_·7H_2_O and 20 g/l glucose) containing 0.1% each of uridine and uracil were used to cultivate *A. oryzae* strains.

### Plasmid construction and transformation

Primers used in this study are listed in Supplemental Table S3. To construct the plasmids for deletion of genes encoding class VI GPCRs, the upstream flanking region (1 kb) was amplified by PCR and cloned into the donor vector of Multi-Site Gateway cloning system (Invitrogen, Carlsbad, CA) using BP recombinase to generate a 5′ entry clone as described (Maruyama and Kitamoto 2008;Mabashi et al. 2006). The downstream flanking region (1 kb) was joined together with the 300-bp proximal sequence of the upstream region by fusion PCR and cloned into another donor vector to generate 3′ entry clone. Then, these entry clones and the destination vector carrying the *pyrG* marker were joined together using the LR recombinase to generate a plasmid for gene disruption. For the complementation analyses, the coding regions of *gprK-1*, *gprK-2*, and *gprR* were amplified by PCR together with the 1-kb upstream regions using the RIB40 genome as a template and inserted into the pUXN-C-EGFP plasmid carrying the *niaD* marker and *egfp* sequence (Mamun et al. 2020) to construct the plasmid for expression of full-length GPCR-EGFP fusion protein. Plasmids for expression of 7-TM domain-EGFP fusion and RGS domain-EGFP fusion proteins were constructed by inverse PCR to eliminate the RGS domain and 7-TM region, respectively. The amplified fragments served for phosphorylation and self-ligation. The EN/AA mutation in the RGS domain was introduced by inverse PCR. The control strain was generated by transforming the host with the empty vector pUXN-C-EGFP. The plasmids were linearized and integrated into the *niaD* locus of the host.

To construct the plasmids for *amyB* promoter-driven expression of GTPase-deficient mutants of Gα, *AogpaA*, *AogpaB*, and *AoganA*, genes were amplified by PCR and then inserted into the pUt-C-EGFP plasmid that was constructed by inserting the *amyB* promoter sequence from the *A. oryzae* genome into pUXN-C-EGFP. The resultant plasmids were used as templates to perform inverse PCR to introduce the Q/L mutation in the conserved DXXGQ sequence of the GTPase domain (Supplemental Fig. S1; Masters et al. 1989). The plasmids thus generated and the vector pUXN-C-EGFP were used for fungal transformation. For disruption of Gα-encoding genes, upstream and downstream flanking sequences, 1 kb each, were amplified by PCR and joined together with the *sC* marker sequence by fusion PCR. The resultant fragments were used to transform the triple gene deletion mutant. The control strain was generated by transforming the triple gene deletion mutant by the *sC* marker-containing plasmid. The plasmids for overexpression of RGS domain-containing proteins and VelB were generated using pUt-C-EGFP. The fragment for deletion of *velB* was generated by joining the upstream and downstream flanking regions (1 kb each) to the *sC* marker. Transformation of *A. oryzae* was done according to the method of Kitamoto (2002).

### Elimination of the *pyrG* marker

Conidia from the *pyrG*^+^ gene disruptant (about 1 × 10^6^) were inoculated onto PD agar plate containing 1 mg 5-fluoroorotic acid (FOA)/ml, 0.5% uridine, and 0.2% uracil and then cultured at 30 °C. After 5–8 days, colonies were transferred to another 5-FOA agar plate supplemented with uridine and uracil. 5-FOA-resistant strains were tested for uridine/uracil auxotrophy. The clones obtained were examined for the absence of *pyrG* marker by colony PCR.

### Phenotypic analyses

Germination rates were measured as previously described with a slight modification (Ni et al. 2005). To examine the growth phenotypes, about 1 × 10^6^ conidia of each strain were inoculated onto a DPY agar plate containing uridine and uracil and cultured for 4 days at 30 °C to check the growth rate and the number of sclerotia formed with three replicate plates per condition.

### Heterologous expression of RGS domains of class VI GPCRs in yeast

GAP activity of the RGS domain in GprK-1, GprK-2, and GprR was indirectly measured as previously described (Chen et al. 2003). First, the RGS domain regions (AoGprK-1, residues 294–560; AoGprK-2, residues 295–562; AoGprR, residues 290–520) were identified through the amino acid sequence analysis. DNA fragments encoding these RGS domain regions were amplified using the forward primer containing the start codon and inserted into the expression vector pYES2 (Invitrogen, Tokyo, Japan) using the In-Fusion HD Cloning kit (Takara bio, Kusatsu, Japan). The resultant plasmids were transformed to *Saccharomyces cerevisiae sst2*∆ null mutant strain YO6055 (BY4741; *MAT*a, *his3*Δ*1*, *leu2*Δ*0*, *met15*Δ*0*, *ura3*Δ*0*, *YLR452c*::*kanMX4*). The transformants were further transformed by the reporter plasmid BYP5232 (https://yeast.nig.ac.jp/yeast/by/PlasmidDetail.jsf?id=5232) that allows the expression of the β-galactosidase reporter under the control of pheromone-responsive *FUS1* promoter. As a control strain, the wild type strain BY4741 was also transformed by BYP5232. Reporter assays were performed as described (Hoffman and Garrison 2002).

### Gene expression analysis

Each strain was inoculated on DPY agar medium containing uridine and uracil and grown for 4 days at 30 °C. The cells were suspended in distilled water containing 0.01% Tween 20, washed twice with water, and then disrupted using multi-bead shocker (Yasui-kikai, Osaka, Japan). Total RNA extraction was performed according to the method described previously (Chomczynski et al. 1993); cDNA was synthesized using PrimeScript™ 1st strand cDNA Synthesis Kit (Takara Bio, Kusatsu, Japan). Finally, qRT-PCR was performed using Thunderbird® SYBR® qPCR Mix (TOYOBO, Osaka, Japan).

## Results

### Identification of class VI GPCRs in *A*. *oryzae*

**Fig. 1:**
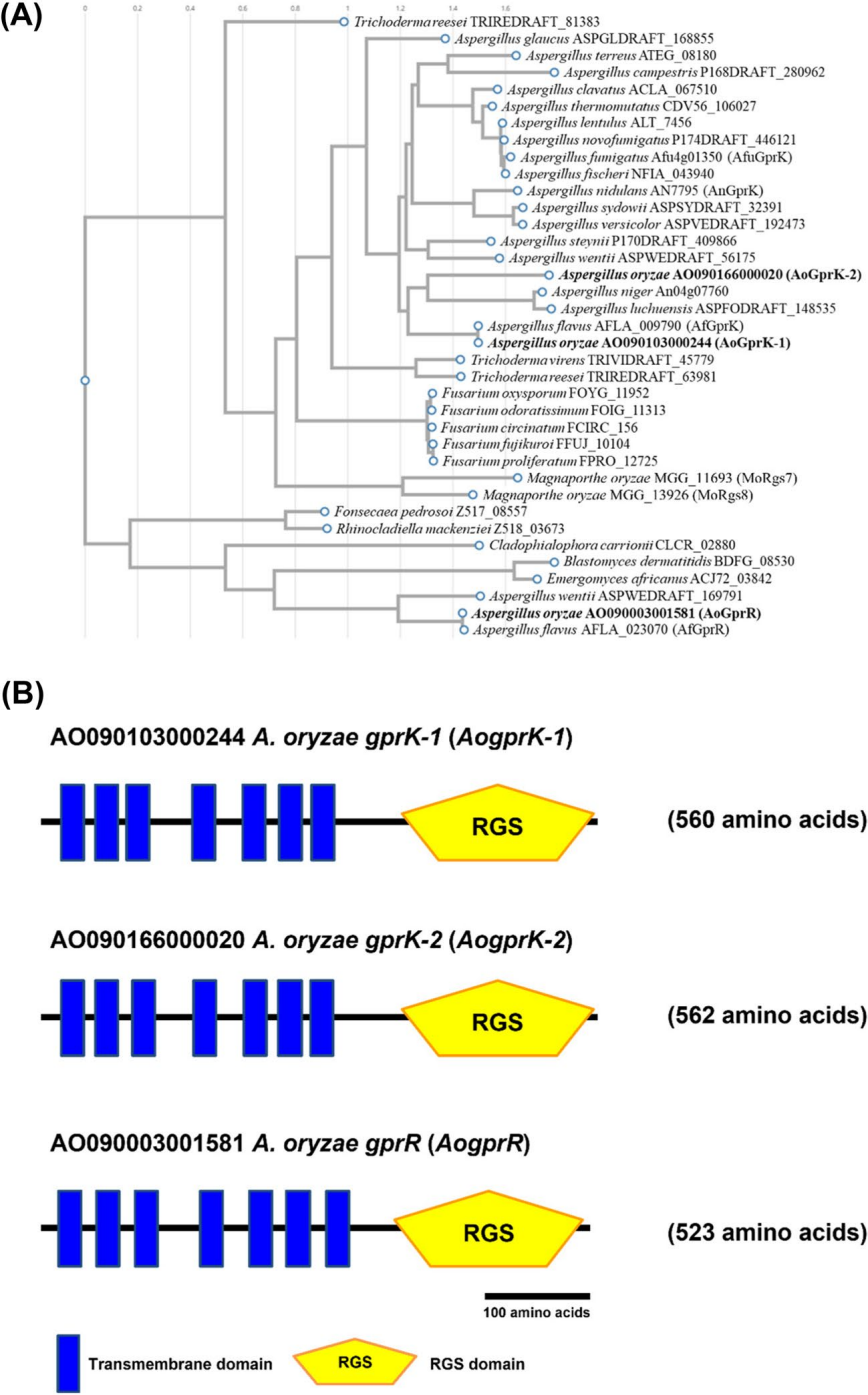
Sequence analysis of class VI GPCRs in filamentous fungi. **A** A phylogenetic tree of class VI GPCRs in filamentous fungi is shown. The protein sequences used are as follows: *A. oryzae* (AO090103000244 (AoGprK-1), AO090166000020 (AoGprK-2), AO090003001581 (AoGprR)), *A. nidulans* (AN7795 (AnGprK)), *A. flavus* (AFLA_009790 (AfGprK), AFLA_023070 (AfGprR)), *A. fumigatus* (Afu4g01350 (AfuGprK)), *A. wentii* (ASPWEDRAFT_56175, ASPWEDRAFT_169791), *A. sydowii* (ASPSYDRAFT_32391), *A. clavatus* (ACLA_067510), *A. luchuensis* (ASPFODRAFT_148535), *E. africanus* (ACJ72_03842), *T. virens* (TRIVIDRAFT_45779), *F. odoratissimum* (FOIG_11313), *A. lentulus* (ALT_7456), *T. reesei* (TRIREDRAFT_81383, TRIREDRAFT_63981), *A. glaucus* (ASPGLDRAFT_168855), *F. proliferatum* (FPRO_12725), *F. circinatum* (FCIRC_156), *B. dermatitidis* (BDFG_08530), *A. terreus* (ATEG_08180), *A. fischeri* (NFIA_043940), *A. steynii* (P170DRAFT_409866), *A. novofumigatus* (P174DRAFT_446121), *A. niger* (An04g07760), *M. oryzae* (MGG_11693 (MoRgs7), MGG_13926 (MoRgs8)), *R. mackenziei* (Z518_03673), *F. fujikuroi* (FFUJ_10104), *A. versicolor* (ASPVEDRAFT_192473), *F. oxysporum* (FOYG_11952), *F. pedrosoi* (Z517_08557), *A. campestris* (P168DRAFT_280962), and *A. thermomutatus* (CDV56_106027). **B** Domain structures of class VI GPCRs in *A. oryzae*. Functional domains were predicted using the Domains/Motifs search in SMART database (Simple Modular Architecture Research Tool, http://smart.embl-heidelberg.de/smart/change_mode.pl)

In the previous studies, a total of 15 GPCR-related genes were identified by in silico analysis of *A. flavus* genome (Affeldt et al. 2014;Lafon et al. 2006). We searched for the *A. oryzae* homologues of class VI GPCRs, *A. flavus* GprK and GprR, and identified two homologues of GprK, AoGprK-1 (AO090103000244) and AoGprK-2 (AO090166000020), and one homologue of GprR, AoGprR (AO090003001581) (Fig. 1 and Supplemental Table S1). Phylogenetic analysis demonstrated that AoGprK-1 and AoGprR are most closely related to GprK and GprR of *A. flavus*, respectively, and separated into two distinct clades (Fig. 1A). Unlike other *Aspergillus* species which harbor only one ortholog of GprK, *A. oryzae* contains one additional ortholog of GprK, AoGprK-2, showing limited similarity to GprK and AoGprK-1. Two orthologs of the GprK family were also found in the rice blast fungus *Magnaporthe oryzae* (Fig. 1A).

### Mutants with multiple gene deletion of class VI GPCR genes display increased formation of sclerotia

In order to examine the physiological roles of class VI GPCRs, single and multiple gene deletion mutants were generated. Deletion mutants were constructed using the *pyrG* marker recycling system (Supplemental Fig. S2; Maruyama and Kitamoto 2008). Transformants were grown on the medium containing 5-FOA to eliminate the *pyrG* marker by homologous recombination, and the deletion of the second gene was conducted. In this way, three single deletion (Δ*AogprK-1*, Δ*AogprK-2*, and Δ*AogprR*), three double deletion (Δ*AogprK-1* Δ*AogprK-2*, Δ*AogprK-1* Δ*AogprR*, and Δ*AogprK-2* Δ*AogprR*), and one triple deletion (Δ*AogprK-1* Δ*AogprK-2* Δ*AogprR*) mutants were obtained.

**Fig. 2:**
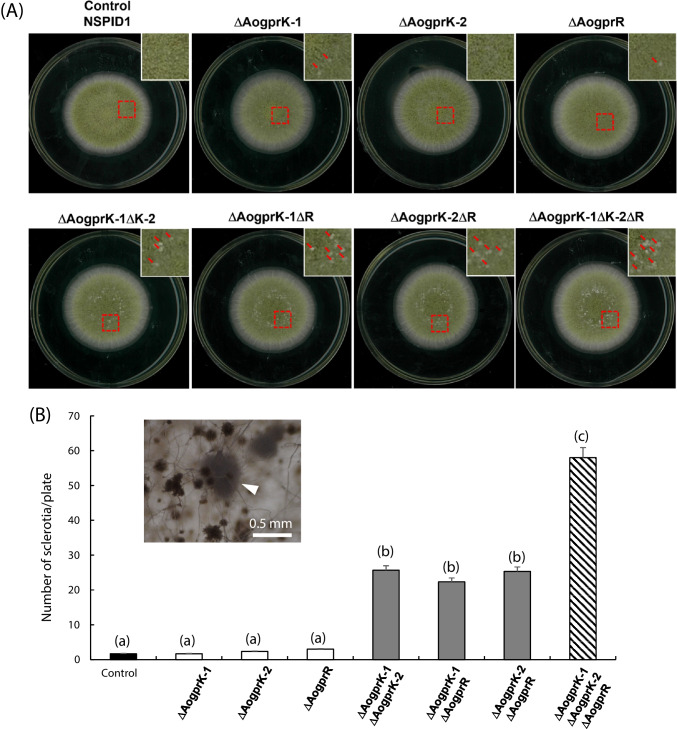
Growth and sclerotia formation of single and multiple gene deletion mutants. **A** Colony photographs of parental (control), single, and multiple gene deletion mutants. Conidial suspensions (10^3^/5 μl) were spotted on the DPY agar plates containing uridine and uracil, incubated for 4 days at 30 °C, and the number of sclerotia was counted. Insets show the enlarged photos, and the red arrows indicate sclerotia. **B** The number of sclerotia in each plate. Error bars represent standard deviation (*n* = 3). Different letters (a–c) indicate that the numbers of sclerotia are statistically different (*p* < 0.05) by Student’s *t*-test. Inset, enlarged photo of the sclerotia formed (arrowhead)

In the previous study of another group, *A. fumigatus gprK* deletion mutant showed no change in radial growth; it formed very faint colonies with highly reduced thallic density and significantly lowered formation of conidiophores compared to the wild type strain (Jung et al. 2016). In contrast, in *A. oryzae*, no change was observed in the conidia formation and colony size in the three single deletion mutants, three double deletion mutants, and one triple deletion mutant compared to the control strain. Interestingly, however, when the sclerotia formation was analyzed, we found that the single deletion mutants produced a slightly increased number of sclerotia, and this phenotype was significantly enhanced in the double and triple deletion mutants, where the triple deletion mutant produced a much higher number of sclerotia than the double deletion mutants (Fig. 2). The size of sclerotia was around 0.5 mm as shown in the inset of Fig. 2B. The images of plates of the control and the triple gene deletion mutant after washing out conidia are shown in Supplemental Fig. S3.

**Fig. 3:**
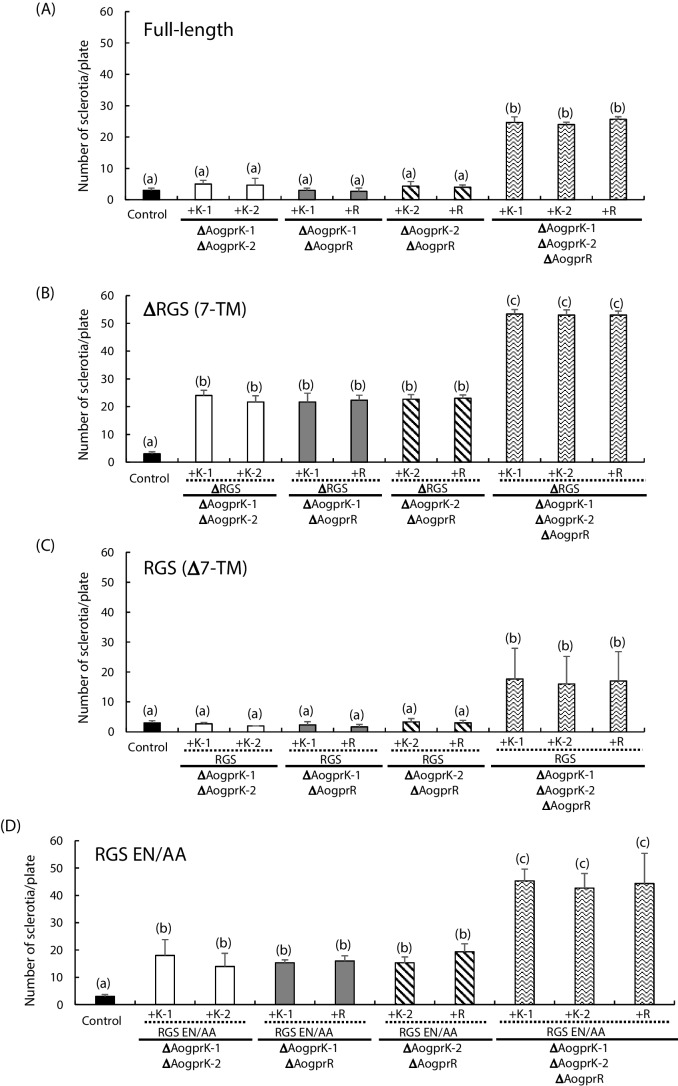
Sclerotia formation in the complemented strains. Conidial suspensions (10^3^/5 μl) were spotted on the DPY agar plates containing uridine and uracil, incubated for 4 days at 30 °C, and the number of sclerotia was counted. The number of sclerotia in the strains complemented by the full-length constructs (**A**), the constructs containing only the 7-TM domain (**B**), the constructs containing only the RGS domain (**C**), and the full-length constructs containing EN/AA mutation in the RGS domains (**D**), respectively, was counted. The genotypes of the host strains are shown in the bottom, and the complemented gene is indicated by “+” (e.g., “+K-1” indicates that the *AogprK-1* was complemented, while “ΔRGS” indicates that only the 7-TM domain was complemented)

To verify that this phenotype was truly due to the lack of class VI GPCRs, complementation analysis was performed by introducing either one of the deleted genes into the double and triple deletion mutants. We used C-terminally EGFP-fused versions of class VI GPCRs in this analysis. As shown in Fig. 3A and Supplemental Fig. S4A, the double deletion mutants complemented by one of the deleted genes produced a significantly reduced number of sclerotia; the level of sclerotia formation was close to that of the single deletion mutants. Also, the triple deletion mutant complemented by one of the deleted genes produced a decreased number of sclerotia, which was similar to the double deletion mutants. No significant difference was observed among AoGprK-1, AoGprK-2, and AoGprR. Thus, it was demonstrated that formation of sclerotia was negatively regulated by class VI GPCRs in *A. oryzae*.

Since class VI GPCRs are composed of two functional domains, i.e., 7-TM and RGS (Fig. 1B), we next asked if either one or both are involved in the regulation of sclerotia formation. To this end, plasmids for expression of 7-TM-EGFP fusion protein lacking the RGS domain and RGS-EGFP fusion protein lacking the 7-TM domain were constructed and transformed into the double and triple deletion mutants. We found that in all the cases, the strains complemented by the constructs lacking the RGS domain failed to show decrease in sclerotia formation. In contrast, the strains complemented by the constructs that contained only the RGS domain produced decreased number of sclerotia, suggesting that the RGS domain, not the 7-TM domain, is responsible for the negative regulation of sclerotia formation (Fig. 3B and C, Supplemental Figs. S4B and S4C). Like the complementation experiments using the full-length constructs, no significant difference was observed among AoGprK-1, AoGprK-2, and AoGprR.

To further examine if the RGS activity of class VI GPCRs, verified in the next section, is solely responsible for the regulation of sclerotia formation, we generated point mutants in which Glu-Asn dyad residues conserved among human and fungal RGS proteins were mutated to Ala-Ala (Supplemental Figs. S5 and S6); it was previously demonstrated that a mutant form of human RGS4 protein with replacement of both Glu87 and Asn88 with Ala loses its GTPase-activating (GAP) activity toward Gαi (Srinivasa et al. 1998). We introduced this replacement in the full-length AoGprK-1, AoGprK-2, and AoGprR and performed the complementation experiment. Unlike the results of complementation experiments using the wild type sequences, forced expression of class VI GPCRs carrying the EN/AA mutation only slightly decreased the number of sclerotia formed in the double and triple deletion mutants (Fig. 3D and Supplemental Fig. S4D). We thus concluded that the RGS function of fungal class VI GPCRs is solely responsible for the negative regulation of sclerotia formation.

Having shown that the RGS domains of class VI GPCRs could complement the phenotype of multiple gene deletion mutants, we next asked if other RGS domain-containing proteins could also regulate sclerotia formation. Other than three class VI GPCRs, *A. oryzae* possesses five RGS domain-containing proteins, RgsA, RgsB, RgsC, RgsD, and FlbA (Lafon et al. 2006). These were individually expressed in the triple gene deletion mutant, and the sclerotia formation was examined. As shown in Supplemental Fig. S7, forced expression of RGS domain-containing proteins resulted in the reduced production of sclerotia, indicating that RGS domains work in a non-specific manner to regulate the formation of sclerotia.

### Heterologous expression and functional analysis of RGS domains of class VI GPCRs in yeast

**Fig. 4:**
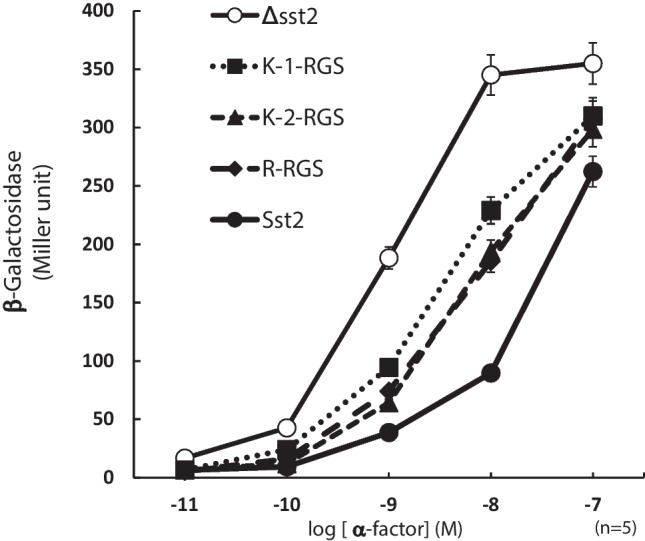
Functional analysis of RGS domains of class VI GPCRs. Response curves for α-factor-induced expression of P_*FUS1*_-*lacZ*. The strains used were Y06055 (Δ*sst2*) harboring the vector (○), RGSs from AoGprK-1 (■), AoGprK-2 (▲), and AoGprR (◆), respectively. As a control, BY4741 (●) expressing endogenous Sst2 was used. Cells grown to early log phase (OD_600_ = 0.4) in the YPG medium were incubated for 2 h in the presence of indicated concentrations of α-factor prior to the assay of β-galactosidase activity

To confirm the RGS activity of fungal class VI GPCRs, an experiment was conducted to examine the pheromone response in yeast. Loss-of-function mutations in *sst2* encoding the archetypal RGS protein of *S. cerevisiae* render haploid *MAT*a yeast cells hypersensitive to α-factor signaling mediated by a canonical GPCR, Ste2 (Dohlman et al. 1996). Like Sst2, it was reported that the RGS domain of *A. thaliana* AtRGS1 could negatively regulate the pheromone pathway (Chen et al. 2003). A similar experimental setting was used to examine the RGS activity of fungal class VI GPCRs. A construct carrying the *lacZ* reporter controlled by the pheromone pathway-specific promoter P_*FUS1*_ was introduced to the *sst2*Δ mutant together with the plasmids for expression of RGS domains of class VI GPCRs, and the β-galactosidase activity was measured. As shown in Fig. 4, expression of RGS domains of class VI GPCRs partially restored the hypersensitivity of *sst2*Δ mutant to α-factor, suggesting that the RGS domains of fungal class VI GPCRs exert GAP activity toward the yeast Gα subunit Gpa1. No noticeable difference was found among RGS domains from AoGprK-1, AoGprK-2, and AoGprR.

### Involvement of Gα subunits, AoGpaA and AoGpaB, in sclerotia formation

**Fig. 5:**
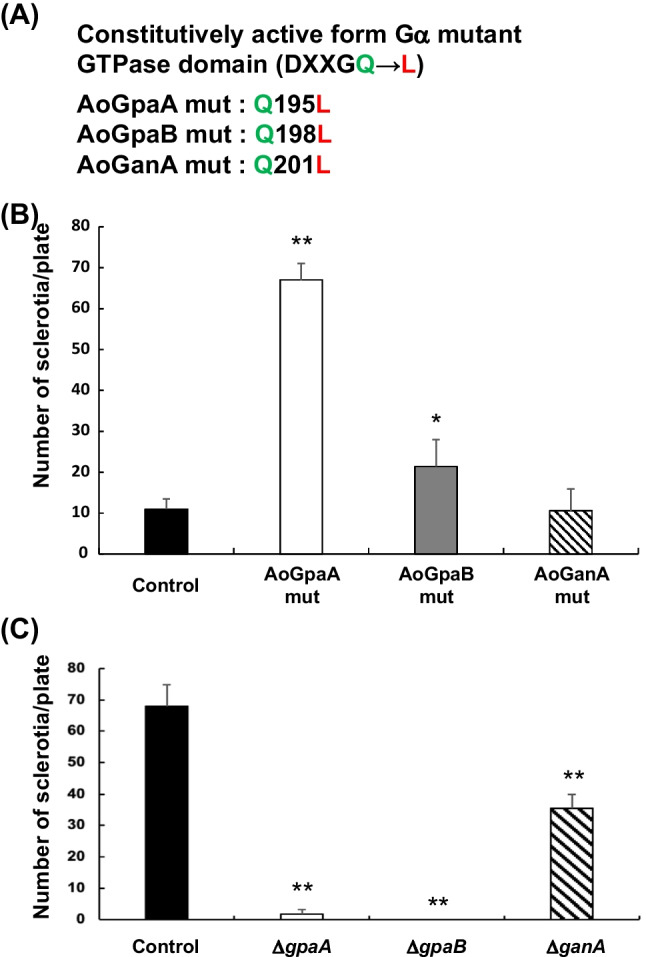
Sclerotia formation in the Gα-overexpressing and -deleted strains. **A** Mutant Gα sequences introduced into the control strain to generate Gα-overexpressing strains used in **B**. **B** Conidial suspensions (10^3^/5 μl) were spotted on the CD agar plates containing methionine and 2% dextrin instead of glucose, incubated for 5 days at 30 °C, and the number of sclerotia was counted. **C** Genes encoding Gα subunits were deleted using the triple gene deletion mutant as a host, and the resultant disruptants and the control strain were examined for sclerotia formation as described in **B**. Error bars represent standard deviation (*n* = 3). * and **, *p* < 0.05 and *p* < 0.01 by Student’s *t*-test, respectively

Since RGS activates the GTPase activity of Gα and inactivates its signaling, the absence of RGS would result in the persisted activation of Gα. We hypothesized that the increased sclerotia formation in the strains with multiple gene deletion was caused by the augmentation of Gα signals. We therefore examined if expression of GTPase-deficient mutant of Gα in *A. oryzae* would lead to increased sclerotia formation. Alignment of three Gαs in *A. oryzae*, AoGpaA, AoGpaB, and AoGanA, with yeast Gpa1 along with human Gαs and Gαi proteins, demonstrated that the amino acid sequences in the guanine nucleotide binding motif (GXGXXGKS; boxed in red in Supplemental Fig. S1) and GTPase domain (DXXGQ; boxed in blue) are highly conserved. It was previously demonstrated that Q-to-L mutation in the GTPase domain decreased the *k*_cat_ of GTPase activity for Gαs by 100-fold (Masters et al. 1989). We generated the strains exogenously expressing the Q-to-L mutant of either AoGpaA, AoGpaB, or AoGanA (Fig. 5A) by transforming the wild type strain, grew them on the dextrin-containing medium to induce the *amyB* promoter-driven expression of Gα mutant, and observed the sclerotia formation. Elevated expression of each Gα subunit was confirmed by qPCR (Supplemental Fig. S8). As shown in Fig. 5B, cells expressing the GTPase-deficient mutants of AoGpaA and AoGpaB, but not AoGanA, exhibited an increased number of sclerotia compared to the control strain. We also generated the strains deleted for the genes encoding Gα subunits using the triple gene deletion mutant as a host and examined the sclerotia formation. As shown in Fig. 5C, increased sclerotia formation in the triple gene deletion mutant was severely impaired by the deletion of *AogpaA* or *AogpaB*. Although it should be noted that the radial growth of the Δ*AogpaA* mutant was slower than that of the other strains (Supplemental Fig. S9), these results collectively demonstrate that AoGpaA and AoGpaB are involved in the regulation of sclerotia formation.

### Expression analysis of genes involved in sclerotia formation

**Fig. 6:**
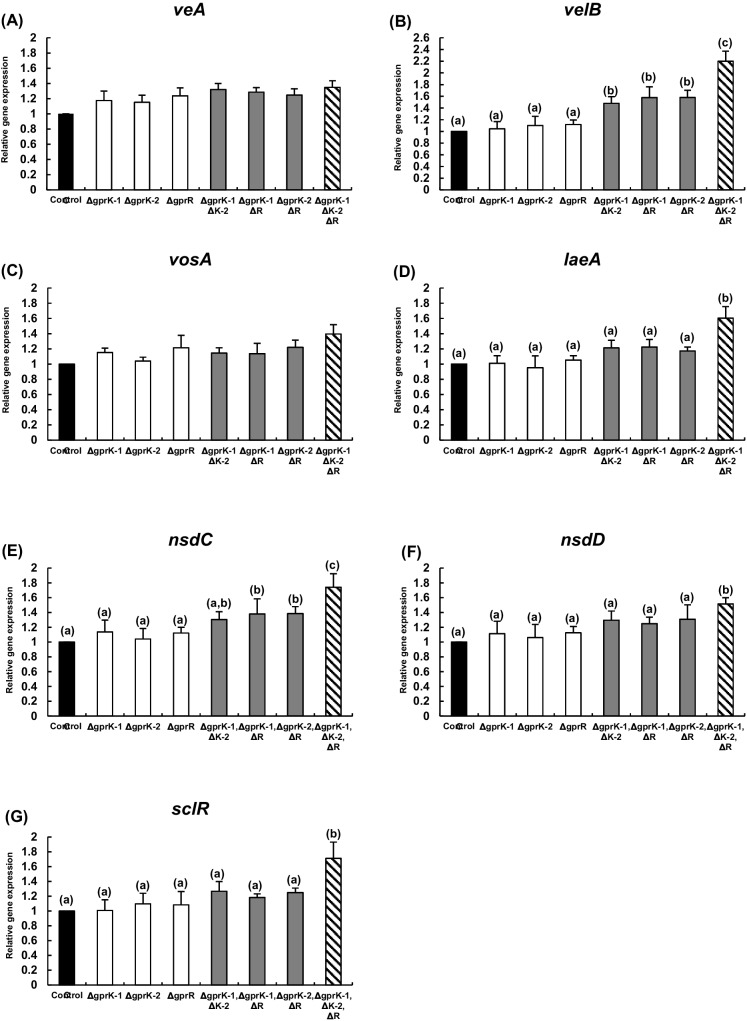
Relative expression levels of velvet complex genes and sclerotia formation-related genes. Relative expression levels of genes encoding the velvet complex and related proteins (*veA* (**A**), *velB* (**B**), *vosA* (**C**), and *laeA* (**D**)), and those related to sclerotia formation (*nsdC* (**E**), *nsdD* (**F**), and *sclR* (**G**)) were measured by qRT-PCR (*n* = 3). Different letters (a–c) indicate that the expression levels are statistically different (*p* < 0.05) by Student’s *t*-test

Sclerotia are considered to be a vestige of the sexual structures, cleistothecia, produced by *Aspergillus* spp. such as *Aspergillus nidulans*. Sclerotia remain dormant or quiescent when the environment is unfavorable but germinate when conditions improve. Various proteins are known to be involved in sclerotia formation, and among them are the velvet complex and related proteins (VeA, VelB, LaeA, and VosA). VeA is involved in the production of conidia, sclerotia, and mycotoxins, while VelB plays multiple roles in the growth, development, and secondary metabolism in *Aspergillus* spp. (Park and Yu 2016). Especially, significantly decreased sclerotia formation was observed in the *veA* or *velB* deletion mutants in *A. flavus* (Duran et al. 2007;Chang et al. 2013). LaeA is a global regulatory protein and forms VeA-LaeA and VelB-VeA-LaeA complexes, which regulate sexual development (Bayram et al. 2008). Like other velvet proteins, VosA also forms various types of complexes such as VosA-VelB, VosA-VosA, and VelB-LaeA-VosA. VosA has been reported as a key player in the spore life cycle in *A. nidulans* and is required for the proper transition from hyphal growth to development including conidiation (Ni and Yu 2007). Besides, it has been reported that *nsdC* and *nsdD* play important roles as sclerotia development genes (Han et al. 2001;Lee et al. 2014) In addition, SclR has also been shown to inhibit asexual development, regulate hyphal morphology, and promote sclerotia formation in *A. oryzae* (Jin et al. 2011). We therefore examined the expression levels of these genes (Fig. 6). As a result, it was demonstrated that the expression levels of some genes were increased in the multiple gene deletion mutants compared to the control strain. Although there was no significant change in the expression of *veA* and *vosA*, the expression of *velB* was significantly enhanced in the double and the triple gene deletion mutants compared to the control strain and the single gene deletion mutants, showing about 2.2-fold increase in the triple gene deletion mutant compared to the control strain (Fig. 6B). The expression of *laeA* was also increased in the triple gene deletion mutant (Fig. 6D). In addition, the sclerotia formation-related gene *nsdC* was upregulated in the two double and the triple gene deletion mutants (Fig. 6E), while the expression of *nsdD* and *sclR* was increased in the triple gene deletion mutant compared to other strains (Fig. 6F and G).

**Fig. 7:**
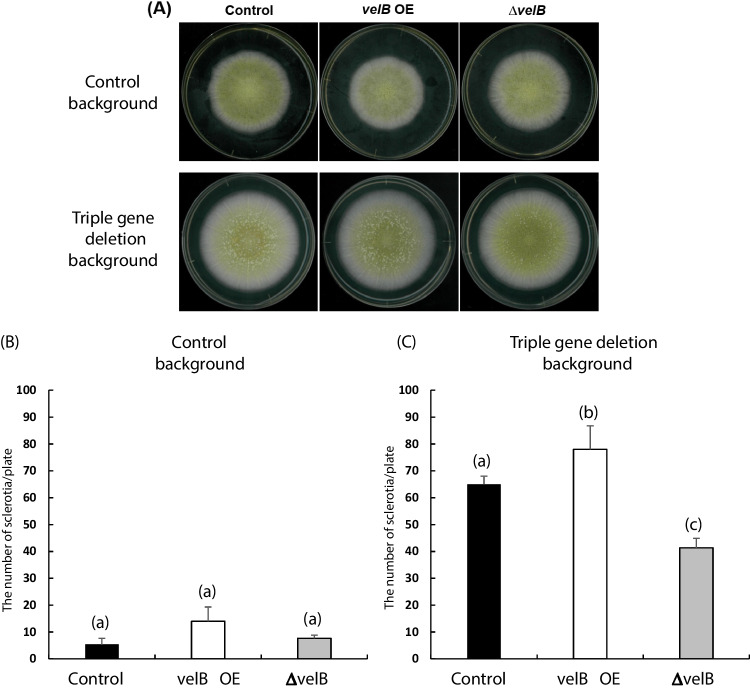
Effects of overexpression and deletion of *velB* on sclerotia formation. Colony photographs of *velB*-overexpression and *velB*-deletion strains generated from the control and triple gene deletion mutants (**A**). Conidial suspensions (10^3^/5 μl) were spotted on the DPY agar plates containing uridine and uracil, incubated for 4 days at 30 °C, and the number of sclerotia was counted. The number of sclerotia from each strain (**B**, **C**). Different letters indicate that the strains derived from the control (**B**) or triple gene deletion backgrounds (**C**) show significant difference (*p* < 0.05) by Student’s *t*-test (*n* = 5)

Since the increased expression of *velB* was observed in the double and triple gene deletion mutants, and the previous studies have shown that the expression of *velB* is highly correlated with sclerotia formation and that the deletion of *velB* results in the inability of the fungus to form sclerotia (Park and Yu 2016), we next asked if overexpression or deletion of *velB* in the control and the triple gene deletion backgrounds affects sclerotia formation. As shown in Fig. 7, overexpression of *velB* caused a slight increase in the number of sclerotia formed in both the control and triple gene deletion backgrounds. More importantly, deletion of *velB* in the triple gene deletion background resulted in the reduced formation of sclerotia. In addition, as shown in Supplemental Fig. S10, the expression of *velB* in the triple gene deletion mutant was decreased when complemented by one of the constructs carrying only the RGS domain of either AoGprK-1, AoGprK-2, or AoGprR. Taken together, these results demonstrate that the *velB* is the key regulator that plays an important role in the increased formation of sclerotia in the multiple gene disruptants and that the class VI GPCRs negatively regulate the expression and function of *velB*.

## Discussion

In this study, we performed phenotypic observation and gene expression analysis to characterize the functions of three class VI GPCRs in *A. oryzae*, AoGprK-1, AoGprK-2, and AoGprR, which exhibit the unique hybrid structure containing the RGS domain at the C terminus of 7-TM domain, distinct from the canonical GPCRs (Fig. 1). Although the deletion of either one of three failed to show any phenotypic alterations, the double and triple gene deletion mutants showed a remarkable phenotype with a significant increase in the number of sclerotia formed (Fig. 2). Complementation analyses demonstrated that the loss of RGS function, but not the 7-TM domain with hypothetical ligand-binding and guanine nucleotide exchange functions, was responsible for the production of increased number of sclerotia in the multiple gene deletion mutants (Fig. 3). Using the pheromone response assay, the RGS domains of class VI GPCRs were shown to exhibit the GAP activity (Fig. 4). Importantly, this is the first experimental evidence showing that the RGS domain of fungal GprK (and GprR) actually displays the GAP activity, which is in line with the result of complementation assay where the EN/AA mutants with a defect in the interaction between RGS and Gα failed to restore the phenotype. Consistent with the involvement of RGS function in the regulation of sclerotia formation, forced expression of active form of Gα subunits, AoGpaA and AoGpaB, increased sclerotia formation (Fig. 5). Finally, *velB* was shown to play a key role in controlling sclerotia formation at the downstream of class VI GPCRs (Figs. 6 and 7). Overall, these results suggest that class VI GPCRs principally serve as RGS that negatively regulates the activity of trimeric G protein(s) in a similar manner to AtRGS1, a GPCR of plant origin with the hybrid structure containing 7-TM and RGS domains.

It is of note that in *A. flavus*, deletion of *fadA*, a counterpart of *AogpaA*, inhibited sclerotia formation, and the forced expression of a *fadA* mutant encoding the inactive form resulted in the production of fewer number of sclerotia than in the wild-type (Xie et al. 2022). Meanwhile, *A. nidulans* forms cellular structures called cleistothecia, which are considered to be the counterparts of sclerotia (Geiser et al. 1996), and it has been reported that deletion of *fadA* in *A. nidulans* severely impaired cleistothecia formation, although the formation of surrounding Hülle cells that are proposed to support the development of cleistothecia was not much affected (Rosen et al. 1999). These results are consistent with our data showing that the expression of active form of AoGpaA resulted in the increased formation of sclerotia. Taken together, it is proposed that in *A. oryzae*, AoGpaA positively regulates sclerotia formation while class VI GPCRs negatively regulate AoGpaA, and the loss of class VI GPCRs causes hyperactivation of AoGpaA, resulting in the enhanced sclerotia formation. Since AoGpaA belongs to adenylate cyclase-inhibiting Gi superfamily (Li et al. 2007), it is of interest to examine the level of cyclic AMP in the multiple gene disruptants.

In this scenario, it is predicted that the class VI GPCRs physically interact with AoGpaA and inactivate it, but our attempt to show direct interaction of class VI GPCRs with AoGpaA as well as AoGpaB and AoGanA through the mating-based split ubiquitin system (mbSUS) (Grefen et al. 2009) failed. Since *A. oryzae* proteins heterologously expressed in *S. cerevisiae* in mbSUS might have been unstable, detecting protein-protein interaction in *A. oryzae* using the bimolecular fluorescence complementation (BiFC) method could be an option. Besides, it has been reported that the single Gα protein in *A. thaliana*, AtGPA1, self-activates through the spontaneous GDP-GTP exchange, and the hybrid-type GPCR AtRGS1 interacts with and inactivates it through the RGS function (Johnston et al. 2007). Therefore, it would be intriguing to examine whether AoGpaA is also a self-activating Gα protein.

The complementation experiments demonstrated that the forced expression of only the RGS domain of *A. oryzae* class VI GPCRs lacking the 7-TM domain was sufficient to restore the phenotype observed in the multiple gene deletion mutant (Fig. 3D). Decrease in the sclerotia formation was also observed when the RGS domain-containing proteins were overexpressed in the triple gene deletion mutant (Supplemental Fig. S7). In contrast, the phenotypes such as increased production of cAMP, abnormal appressorium formation, and decreased pathogenicity observed in the Δ*Morgs7* mutant of *M. oryzae* were fully restored only when the full-length construct was complemented; only the partial restoration of phenotypes was observed by expressing the RGS domain, and when the 7-TM domain was complemented, no recovery of phenotypes was observed (Li et al. 2019a). Since the authors also showed that both RGS and 7-TM domains bound to MoMagA, one of the three Gα subunits in *M. oryzae*, the 7-TM domain might have a role in the interaction of MoRgs7 with Gα, which could explain the reason why the expression of the RGS domain only partially recovered the phenotype. Another study also demonstrated that the full functionality of GprK in *A. fumigatus* required both the 7-TM and the RGS domains (Jung et al. 2016). In contrast, no obvious functional role was assigned to the 7-TM domain of *A. oryzae* class VI GPCRs; as stated above, increased formation of sclerotia in the triple gene deletion mutant was restored to the level of the double gene disruptants by expressing one of the three RGS domains from class VI GPCRs. Although class VI GPCR-EGFP fusion proteins localized to the plasma membrane (data not shown), this result suggests that anchoring the RGS domain to the plasma membrane is not essential for the regulation of sclerotia formation by class VI GPCRs. In this sense, the precise localization of class VI RGS domain lacking the 7-TM domain as well as the five RGS domain-containing proteins needs to be examined.

In the multiple gene disruptants, especially the triple gene deletion mutant, the expression levels of genes encoding the velvet complex and those with sclerotia-related function were higher than in the control strain. Especially the expression of *velB* correlated well with sclerotia formation in that the increase was observed in the double and triple gene deletion mutants. In *Aspergillus* spp., VelB plays key roles in the growth, development, and secondary metabolism, and deletion of *velB* results in significant decrease in sclerotia formation in *A. flavus* (Park and Yu 2016;Duran et al. 2007;Chang et al. 2013). In line with this, our results showed that deletion of *velB* in the triple gene knockout background caused a significant decrease in the number of sclerotia formed. However, the extent of increase of *velB* expression in the triple gene deletion mutant was much lower compared to that of sclerotia formation, and *velB* deletion did not fully abolish sclerotia formation. These results suggest that class VI GPCRs control sclerotia formation through VelB-dependent and VelB-independent pathways. Considering that expression of active form of AoGpaB also stimulated sclerotia formation, albeit to a lesser extent than AoGpaA, multiple pathways might be involved in the control of sclerotia formation. In-depth analysis of the signaling pathway that leads to sclerotia formation as well as the search for the ligand(s) for class VI GPCRs are of central importance in the future studies.

## Supplementary information

ESM 1

## Data Availability

All data generated or analyzed during this study are included in this published article and its supplementary information files.
