# Supplementary material for: Class VI G protein-coupled receptors in Aspergillus oryzae regulate sclerotia formation through GTPase-activating activity
Source: Appl Microbiol Biotechnol. 2024 Jan 17;108:141. doi: 10.1007/s00253-023-12862-0 (PMC10794492; doi:10.1007/s00253-023-12862-0)
Supplement: Supplementary file 1 [file 253_2023_12862_MOESM1_ESM.pdf]

Supplemental Material

Journal
Applied Microbiology and Biotechnology

Title
Class VI G protein-coupled receptors in *Aspergillus oryzae* regulate sclerotia formation through GTPase-activating activity

Authors
Dong Min Kim[1], Itsuki Sakamoto[1], and Manabu Arioka[1, 2, *]

Affiliation
[a]Department of Biotechnology, The University of Tokyo, Japan
[b]Collaborative Research Institute for Innovative Microbiology (CRIIM), The University of Tokyo, Japan

*Corresponding author
Department of Biotechnology, The University of Tokyo
1-1-1 Yayoi, Bunkyo-ku, Tokyo 113-8657, Japan
Tel.: +81-3-5841-5163; Fax: +81-3-5841-8033
E-mail: arioka@mail.ecc.u-tokyo.ac.jp

Table S1. Overview of *A. oryzae* GPCRs and potential roles of their orthologues in *A. flavus*

Class	Gene	Amino acids	Predicted roles	Conserved domain	Observed roles in *A. flavus* (Affeldt et al. 2014)
I	*AogprA*	374	Mating	STE2 GPCR (*S. cerevisiae* pheromone receptor)	Germination; AF repression; carbon source sensing; oxylipin sensing
II	*AogprB*	465	Mating	STE3 GPCR (*S. cerevisiae* pheromone receptor)	Germination; quorum sensing; MeJA sensing
III	*AogprC*	444	Glucose sensing	Git3; Gi3C (*Schizosaccharo-myces pombe* glucose receptor)	Germination; carbon and nitrogen sensing; 13(S)-HpODE sensing
	AogprD	415	Nitrogen sensing		Nitrogen and 13(S)-HpODE sensing; ROS, cell wall, acidic pH stress response
IV	*AogprF*	300	Nitrogen sensing	PQ loop repeat (*Schizosaccharo-myces pombe* nitrogen sensor)	Light sensing; quorum sensing; acidic pH stress response; oxylipin sensing
	AogprG	426			ROS and acidic pH stress responses; oxylipin sensing
	AogprJ	322			Germination; carbon and 13(S)-HpODE sensing
	AogprS	266			Germination; cell wall stress response; MeJA sensing
V	*AogprH*	428	Methionine sensing	Secretin family (signal through cAMP pathways)	Germination; ROS stress response
VI	*AogprK-1*	560	Unknown	RGS domain (regulator of G protein signaling)	Germination; carbon, MeJA sensing; cell wall, osmotic, and acidic stress response
	AogprK-2	562			
	AogprR	523			Germination; light; carbon and nitrogen sensing; osmotic and alkaline pH stress responses; lipid and oxylipin sensing
VII	*AogprM*	490	Unknown	No conserved domains	Carbon, MeJA sensing, osmotic and pH stress responses
VIII	*AogprO*	282	Unknown	Hemolysin III related (broad range of ligands)	Oxylipin sensing
	AogprP	508			Germination; AF repression; carbon sensing; Oxylipin sensing
IX	*AonopA*	312	Light sensing	Bacteriorhodopsin-like (photoreactive)	Unknown

AF, aflatoxin; MeJA, methyl jasmonate; 13(S)-HpODE, 13S-hydroperoxy-9Z,11E-octadecadienoic acid;

ROS, reactive oxygen species

Affeldt KJ, Carrig J, Amare M, Keller NP (2014)

Global survey of canonical *Aspergillus flavus* G protein-coupled receptors. mBio 5: 1501. doi: 10.1128/mBio.01501-14

Table S2. Strain list

***A. oryzae* strain list**

Strain	Host	Genotype	Reference
NSPlD1 (Control)	NSlD	niaD$^-$ sC$^-$ adeA$^-$ ΔargB::adeA$^-$ ΔligD::argB ΔpyrG::adeA	Maruyama and Kitamoto (2008)
ΔAoGprK-1	NSPlD1	niaD$^-$ sC$^-$ adeA$^-$ ΔargB::adeA$^-$ ΔligD::argB ΔpyrG::adeA ΔAogprK-1	This study
ΔAoGprK-2	NSPlD1	niaD$^-$ sC$^-$ adeA$^-$ ΔargB::adeA$^-$ ΔligD::argB ΔpyrG::adeA ΔAogprK-2	This study
ΔAoGprR	NSPlD1	niaD$^-$ sC$^-$ adeA$^-$ ΔargB::adeA$^-$ ΔligD::argB ΔpyrG::adeA ΔAogprR	This study
ΔAoGprK-1ΔK-2	ΔAoGprK-1	niaD$^-$ sC$^-$ adeA$^-$ ΔargB::adeA$^-$ ΔligD::argB ΔpyrG::adeA ΔAogprK-1 ΔAogprK-2	This study
ΔAoGprK-1ΔR	ΔAoGprK-1	niaD$^-$ sC$^-$ adeA$^-$ ΔargB::adeA$^-$ ΔligD::argB ΔpyrG::adeA ΔAogprK-1 ΔAogprR	This study
ΔAoGprK-2ΔR	ΔAoGprR	niaD$^-$ sC$^-$ adeA$^-$ ΔargB::adeA$^-$ ΔligD::argB ΔpyrG::adeA ΔAogprK-2 ΔAogprR	This study
ΔAoGprK-1ΔK-2ΔR	ΔAoGprK-1ΔK-2	niaD$^-$ sC$^-$ adeA$^-$ ΔargB::adeA$^-$ ΔligD::argB ΔpyrG::adeA ΔAogprK-1 ΔAogprK-2 ΔAogprR	This study
ΔAoGprK-1+AoGprK-1	ΔAoGprK-1	niaD$^-$ sC$^-$ adeA$^-$ ΔargB::adeA$^-$ ΔligD::argB ΔpyrG::adeA ΔAogprK-1 niaD$^-$::(AogprK-1-egfp)	This study
ΔAoGprK-2+AoGprK-2	ΔAoGprK-2	niaD$^-$ sC$^-$ adeA$^-$ ΔargB::adeA$^-$ ΔligD::argB ΔpyrG::adeA ΔAogprK-2 niaD$^-$::(AogprK-2-egfp)	This study
ΔAoGprR+AoGprR	ΔAoGprR	niaD$^-$ sC$^-$ adeA$^-$ ΔargB::adeA$^-$ ΔligD::argB ΔpyrG::adeA ΔAogprR niaD$^-$::(AogprR-egfp)	This study
ΔAoGprK-1ΔK-2+AoGprK-1	ΔAoGprK-1ΔK-2	niaD$^-$ sC$^-$ adeA$^-$ ΔargB::adeA$^-$ ΔligD::argB ΔpyrG::adeA ΔAogprK-1 ΔAogprK-2 niaD$^-$::(AogprK-1-egfp)	This study
ΔAoGprK-1ΔK-2+AoGprK-2	ΔAoGprK-1ΔK-2	niaD$^-$ sC$^-$ adeA$^-$ ΔargB::adeA$^-$ ΔligD::argB ΔpyrG::adeA ΔAogprK-1 ΔAogprK-2 niaD$^-$::(AogprK-2-egfp)	This study
ΔAoGprK-1ΔR+AoGprK-1	ΔAoGprK-1ΔR	niaD$^-$ sC$^-$ adeA$^-$ ΔargB::adeA$^-$ ΔligD::argB ΔpyrG::adeA ΔAogprK-1 ΔAogprR niaD$^-$::(AogprK-1-egfp)	This study
ΔAoGprK-1ΔR+AoGprR	ΔAoGprK-1ΔR	niaD$^-$ sC$^-$ adeA$^-$ ΔargB::adeA$^-$ ΔligD::argB ΔpyrG::adeA ΔAogprK-1 ΔAogprR niaD$^-$::(AogprR-egfp)	This study
ΔAoGprK-2ΔR+AoGprK-2	ΔAoGprK-2ΔR	niaD$^-$ sC$^-$ adeA$^-$ ΔargB::adeA$^-$ ΔligD::argB ΔpyrG::adeA ΔAogprK-2 ΔAogprR niaD$^-$::(AogprK-2-egfp)	This study
ΔAoGprK-2ΔR+AoGprR	ΔAoGprK-2ΔR	niaD$^-$ sC$^-$ adeA$^-$ ΔargB::adeA$^-$ ΔligD::argB ΔpyrG::adeA ΔAogprK-2 ΔAogprR niaD$^-$::(AogprR-egfp)	This study
ΔAoGprK-1ΔK-2ΔR+AoGprK-1	ΔAoGprK-1ΔK-2ΔR	niaD$^-$ sC$^-$ adeA$^-$ ΔargB::adeA$^-$ ΔligD::argB ΔpyrG::adeA ΔAogprK-1 ΔAogprK-2 ΔAogprR niaD$^-$::(AogprK-1-egfp)	This study
ΔAoGprK-1ΔK-2ΔR+AoGprK-2	ΔAoGprK-1ΔK-2ΔR	niaD$^-$ sC$^-$ adeA$^-$ ΔargB::adeA$^-$ ΔligD::argB ΔpyrG::adeA ΔAogprK-1 ΔAogprK-2 ΔAogprR niaD$^-$::(AogprK-2-egfp)	This study
ΔAoGprK-1ΔK-2ΔR+AoGprR	ΔAoGprK-1ΔK-2ΔR	niaD$^-$ sC$^-$ adeA$^-$ ΔargB::adeA$^-$ ΔligD::argB ΔpyrG::adeA ΔAogprK-1 ΔAogprK-2 ΔAogprR niaD$^-$::(AogprR-egfp)	This study
ΔAoGprK-1+AoGprK-1 (ΔRGS)	ΔAoGprK-1	niaD$^-$ sC$^-$ adeA$^-$ ΔargB::adeA$^-$ ΔligD::argB ΔpyrG::adeA ΔAogprK-1 niaD$^-$::(AogprK-1 ΔRGS-egfp)	This study
ΔAoGprK-2+AoGprK-2 (ΔRGS)	ΔAoGprK-2	niaD$^-$ sC$^-$ adeA$^-$ ΔargB::adeA$^-$ ΔligD::argB ΔpyrG::adeA ΔAogprK-2 niaD$^-$::(AogprK-2 ΔRGS-egfp)	This study
ΔAoGprR+AoGprR (ΔRGS)	ΔAoGprR	niaD$^-$ sC$^-$ adeA$^-$ ΔargB::adeA$^-$ ΔligD::argB ΔpyrG::adeA ΔAogprR niaD$^-$::(AogprR ΔRGS-egfp)	This study
ΔAoGprK-1ΔK-2+AoGprK-1 (ΔRGS)	ΔAoGprK-1ΔK-2	niaD$^-$ sC$^-$ adeA$^-$ ΔargB::adeA$^-$ ΔligD::argB ΔpyrG::adeA ΔAogprK-1 ΔAogprK-2 niaD$^-$::(AogprK-1 ΔRGS-egfp)	This study
ΔAoGprK-1ΔK-2+AoGprK-2 (ΔRGS)	ΔAoGprK-1ΔK-2	niaD$^-$ sC$^-$ adeA$^-$ ΔargB::adeA$^-$ ΔligD::argB ΔpyrG::adeA ΔAogprK-1 ΔAogprK-2 niaD$^-$::(AogprK-2 ΔRGS-egfp)	This study
ΔAoGprK-1ΔR+AoGprK-1 (ΔRGS)	ΔAoGprK-1ΔR	niaD$^-$ sC$^-$ adeA$^-$ ΔargB::adeA$^-$ ΔligD::argB ΔpyrG::adeA ΔAogprK-1 ΔAogprR niaD$^-$::(AogprK-1 ΔRGS-egfp)	This study
ΔAoGprK-1ΔR+AoGprR (ΔRGS)	ΔAoGprK-1ΔR	niaD$^-$ sC$^-$ adeA$^-$ ΔargB::adeA$^-$ ΔligD::argB ΔpyrG::adeA ΔAogprK-1 ΔAogprR niaD$^-$::(AogprR ΔRGS-egfp)	This study
ΔAoGprK-2ΔR+AoGprK-2 (ΔRGS)	ΔAoGprK-2ΔR	niaD$^-$ sC$^-$ adeA$^-$ ΔargB::adeA$^-$ ΔligD::argB ΔpyrG::adeA ΔAogprK-2 ΔAogprR niaD$^-$::(AogprK-2 ΔRGS-egfp)	This study
ΔAoGprK-2ΔR+AoGprR (ΔRGS)	ΔAoGprK-2ΔR	niaD$^-$ sC$^-$ adeA$^-$ ΔargB::adeA$^-$ ΔligD::argB ΔpyrG::adeA ΔAogprK-2 ΔAogprR niaD$^-$::(AogprR ΔRGS-egfp)	This study
ΔAoGprK-1ΔK-2ΔR+AoGprK-1 (ΔRGS)	ΔAoGprK-1ΔK-2ΔR	niaD$^-$ sC$^-$ adeA$^-$ ΔargB::adeA$^-$ ΔligD::argB ΔpyrG::adeA ΔAogprK-1 ΔAogprK-2 ΔAogprR niaD$^-$::(AogprK-1 ΔRGS-egfp)	This study
ΔAoGprK-1ΔK-2ΔR+AoGprK-2 (ΔRGS)	ΔAoGprK-1ΔK-2ΔR	niaD$^-$ sC$^-$ adeA$^-$ ΔargB::adeA$^-$ ΔligD::argB ΔpyrG::adeA ΔAogprK-1 ΔAogprK-2 ΔAogprR niaD$^-$::(AogprK-2 ΔRGS-egfp)	This study
ΔAoGprK-1ΔK-2ΔR+AoGprR (ΔRGS)	ΔAoGprK-1ΔK-2ΔR	niaD$^-$ sC$^-$ adeA$^-$ ΔargB::adeA$^-$ ΔligD::argB ΔpyrG::adeA ΔAogprK-1 ΔAogprK-2 ΔAogprR niaD$^-$::(AogprR ΔRGS-egfp)	This study
ΔAoGprK-1+AoGprK-1-RGS	ΔAoGprK-1	niaD$^-$ sC$^-$ adeA$^-$ ΔargB::adeA$^-$ ΔligD::argB ΔpyrG::adeA ΔAogprK-1 niaD$^-$::(AogprK-1 RGS-egfp)	This study
ΔAoGprK-2+AoGprK-2-RGS	ΔAoGprK-2	niaD$^-$ sC$^-$ adeA$^-$ ΔargB::adeA$^-$ ΔligD::argB ΔpyrG::adeA ΔAogprK-2 niaD$^-$::(AogprK-2 RGS-egfp)	This study
ΔAoGprR+AoGprR-RGS	ΔAoGprR	niaD$^-$ sC$^-$ adeA$^-$ ΔargB::adeA$^-$ ΔligD::argB ΔpyrG::adeA ΔAogprR niaD$^-$::(AogprR RGS-egfp)	This study
ΔAoGprK-1ΔK-2+AoGprK-1-RGS	ΔAoGprK-1ΔK-2	niaD$^-$ sC$^-$ adeA$^-$ ΔargB::adeA$^-$ ΔligD::argB ΔpyrG::adeA ΔAogprK-1 ΔAogprK-2 niaD$^-$::(AogprK-1 RGS-egfp)	This study
ΔAoGprK-1ΔK-2+AoGprK-2-RGS	ΔAoGprK-1ΔK-2	niaD$^-$ sC$^-$ adeA$^-$ ΔargB::adeA$^-$ ΔligD::argB ΔpyrG::adeA ΔAogprK-1 ΔAogprK-2 niaD$^-$::(AogprK-2 RGS-egfp)	This study
ΔAoGprK-1ΔR+AoGprK-1-RGS	ΔAoGprK-1ΔR	niaD$^-$ sC$^-$ adeA$^-$ ΔargB::adeA$^-$ ΔligD::argB ΔpyrG::adeA ΔAogprK-1 ΔAogprR niaD$^-$::(AogprK-1 RGS-egfp)	This study
ΔAoGprK-1ΔR+AoGprR-RGS	ΔAoGprK-1ΔR	niaD$^-$ sC$^-$ adeA$^-$ ΔargB::adeA$^-$ ΔligD::argB ΔpyrG::adeA ΔAogprK-1 ΔAogprR niaD$^-$::(AogprR RGS-egfp)	This study
ΔAoGprK-2ΔR+AoGprK-2-RGS	ΔAoGprK-2ΔR	niaD$^-$ sC$^-$ adeA$^-$ ΔargB::adeA$^-$ ΔligD::argB ΔpyrG::adeA ΔAogprK-2 ΔAogprR niaD$^-$::(AogprK-2 RGS-egfp)	This study
ΔAoGprK-2ΔR+AoGprR-RGS	ΔAoGprK-2ΔR	niaD$^-$ sC$^-$ adeA$^-$ ΔargB::adeA$^-$ ΔligD::argB ΔpyrG::adeA ΔAogprK-2 ΔAogprR niaD$^-$::(AogprR RGS-egfp)	This study
ΔAoGprK-1ΔK-2ΔR+AoGprK-1-RGS	ΔAoGprK-1ΔK-2ΔR	niaD$^-$ sC$^-$ adeA$^-$ ΔargB::adeA$^-$ ΔligD::argB ΔpyrG::adeA ΔAogprK-1 ΔAogprK-2 ΔAogprR niaD$^-$::(AogprK-1 RGS-egfp)	This study
ΔAoGprK-1ΔK-2ΔR+AoGprK-2-RGS	ΔAoGprK-1ΔK-2ΔR	niaD$^-$ sC$^-$ adeA$^-$ ΔargB::adeA$^-$ ΔligD::argB ΔpyrG::adeA ΔAogprK-1 ΔAogprK-2 ΔAogprR niaD$^-$::(AogprK-2 RGS-egfp)	This study
ΔAoGprK-1ΔK-2ΔR+AoGprR-RGS	ΔAoGprK-1ΔK-2ΔR	niaD$^-$ sC$^-$ adeA$^-$ ΔargB::adeA$^-$ ΔligD::argB ΔpyrG::adeA ΔAogprK-1 ΔAogprK-2 ΔAogprR niaD$^-$::(AogprR RGS-egfp)	This study
ΔAoGprK-1+EN/AA AoGprK-1	ΔAoGprK-1	niaD$^-$ sC$^-$ adeA$^-$ ΔargB::adeA$^-$ ΔligD::argB ΔpyrG::adeA ΔAogprK-1 niaD$^-$::(AogprK-1 EN/AA RGS-egfp)	This study
ΔAoGprK-2+EN/AA AoGprK-2	ΔAoGprK-2	niaD$^-$ sC$^-$ adeA$^-$ ΔargB::adeA$^-$ ΔligD::argB ΔpyrG::adeA ΔAogprK-2 niaD$^-$::(AogprK-2 EN/AA RGS-egfp)	This study
ΔAoGprR+EN/AA AoGprR	ΔAoGprR	niaD$^-$ sC$^-$ adeA$^-$ ΔargB::adeA$^-$ ΔligD::argB ΔpyrG::adeA ΔAogprR niaD$^-$::(AogprR EN/AA RGS-egfp)	This study
ΔAoGprK-1ΔK-2+ EN/AA AoGprK-1	ΔAoGprK-1ΔK-2	niaD$^-$ sC$^-$ adeA$^-$ ΔargB::adeA$^-$ ΔligD::argB ΔpyrG::adeA ΔAogprK-1 ΔAogprK-2 niaD$^-$::(AogprK-1 EN/AA RGS-egfp)	This study
ΔAoGprK-1ΔK-2+EN/AA AoGprK-2	ΔAoGprK-1ΔK-2	niaD$^-$ sC$^-$ adeA$^-$ ΔargB::adeA$^-$ ΔligD::argB ΔpyrG::adeA ΔAogprK-1 ΔAogprK-2 niaD$^-$::(AogprK-2 EN/AA RGS-egfp)	This study
ΔAoGprK-1ΔR+EN/AA AoGprK-1	ΔAoGprK-1ΔR	niaD$^-$ sC$^-$ adeA$^-$ ΔargB::adeA$^-$ ΔligD::argB ΔpyrG::adeA ΔAogprK-1 ΔAogprR niaD$^-$::(AogprK-1 EN/AA RGS-egfp)	This study
ΔAoGprK-1ΔR+EN/AA AoGprR	ΔAoGprK-1ΔR	niaD$^-$ sC$^-$ adeA$^-$ ΔargB::adeA$^-$ ΔligD::argB ΔpyrG::adeA ΔAogprK-1 ΔAogprR niaD$^-$::(AogprR EN/AA RGS-egfp)	This study
ΔAoGprK-2ΔR+EN/AA AoGprK-2	ΔAoGprK-2ΔR	niaD$^-$ sC$^-$ adeA$^-$ ΔargB::adeA$^-$ ΔligD::argB ΔpyrG::adeA ΔAogprK-2 ΔAogprR niaD$^-$::(AogprK-2 EN/AA RGS-egfp)	This study
ΔAoGprK-2ΔR+EN/AA AoGprR	ΔAoGprK-2ΔR	niaD$^-$ sC$^-$ adeA$^-$ ΔargB::adeA$^-$ ΔligD::argB ΔpyrG::adeA ΔAogprK-2 ΔAogprR niaD$^-$::(AogprR EN/AA RGS-egfp)	This study
ΔAoGprK-1ΔK-2ΔR+EN/AA AoGprK-1	ΔAoGprK-1ΔK-2ΔR	niaD$^-$ sC$^-$ adeA$^-$ ΔargB::adeA$^-$ ΔligD::argB ΔpyrG::adeA ΔAogprK-1 ΔAogprK-2 ΔAogprR niaD$^-$::(AogprK-1 EN/AA RGS-egfp)	This study

ΔAoGprK-1ΔK-2ΔR+EN/AA AoGprK-2	ΔAoGprK-1ΔK-2ΔR	*niaD*$^{-}$ *sC*$^{-}$ *adeA*$^{-}$ *ΔargB::adeA*$^{-}$ *ΔligD::argB ΔpyrG::adeA ΔAogprK-1 ΔAogprK-2 ΔAogprR niaD*$^{-}$::(*AogprK-2* EN/AA *RGS-egfp*)	This study
ΔAoGprK-1ΔK-2ΔR+EN/AA AoGprR	ΔAoGprK-1ΔK-2ΔR	*niaD*$^{-}$ *sC*$^{-}$ *adeA*$^{-}$ *ΔargB::adeA*$^{-}$ *ΔligD::argB ΔpyrG::adeA ΔAogprK-1 ΔAogprK-2 ΔAogprR niaD*$^{-}$::(*AogprR* EN/AA *RGS-egfp*)	This study
NSRku70-1-1A	NSRku70-1-1	*niaD*$^{-}$ *sC*$^{-}$ *adeA*$^{-}$ *ΔargB::adeA*$^{-}$ *Δku70::arg adeA*	Higuchi et al. (2009)
SK-1	NSRku70-1-1	*niaD*$^{-}$ *sC*$^{-}$ *adeA*$^{-}$ *ΔargB::adeA*$^{-}$ *Δku70::arg adeA niaD*$^{-}$::(P*amyB niaD*)	
pUt-gpaA Q/L mut	NSRku70-1-1A	*niaD*$^{-}$ *sC*$^{-}$ *adeA*$^{-}$ *ΔargB::adeA*$^{-}$ *Δku70::arg adeA niaD*$^{-}$::(P*amyB-AogpaB* QL mut *niaD*)	This study
pUt-gpaB Q/L mut	NSRku70-1-1A	*niaD*$^{-}$ *sC*$^{-}$ *adeA*$^{-}$ *ΔargB::adeA*$^{-}$ *Δku70::arg adeA niaD*$^{-}$::(P*amyB-AogpaB* QL mut *niaD*)	This study
pUt-ganA Q/L mut	NSRku70-1-1A	*niaD*$^{-}$ *sC*$^{-}$ *adeA*$^{-}$ *ΔargB::adeA*$^{-}$ *Δku70::arg adeA niaD*$^{-}$::(P*amyB-AoganA* QL mut *niaD*)	This study
Δ3 sC	ΔAoGprK-1ΔK-2ΔR	*niaD*$^{-}$ *sC*$^{-}$ *adeA*$^{-}$ *ΔargB::adeA*$^{-}$ *ΔligD::argB ΔpyrG::adeA ΔAogprK-1 ΔAogprK-2 ΔAogprR* pisCA (*sC*)	This study
Δ3 ΔgpaA	ΔAoGprK-1ΔK-2ΔR	*niaD*$^{-}$ *sC*$^{-}$ *adeA*$^{-}$ *ΔargB::adeA*$^{-}$ *ΔligD::argB ΔpyrG::adeA ΔAogprK-1 ΔAogprK-2 ΔAogprR AogpaA::sC*	This study
Δ3 ΔgpaB	ΔAoGprK-1ΔK-2ΔR	*niaD*$^{-}$ *sC*$^{-}$ *adeA*$^{-}$ *ΔargB::adeA*$^{-}$ *ΔligD::argB ΔpyrG::adeA ΔAogprK-1 ΔAogprK-2 ΔAogprR AogpaB::sC*	This study
Δ3 ΔganA	ΔAoGprK-1ΔK-2ΔR	*niaD*$^{-}$ *sC*$^{-}$ *adeA*$^{-}$ *ΔargB::adeA*$^{-}$ *ΔligD::argB ΔpyrG::adeA ΔAogprK-1 ΔAogprK-2 ΔAogprR AoganA::sC*	This study
NSPlD1 velB OE	NSlD	*niaD*$^{-}$ *sC*$^{-}$ *adeA*$^{-}$ *ΔargB::adeA*$^{-}$ *ΔligD::argB ΔpyrG::adeA niaD*$^{-}$::(*PamyB-AovelB niaD*)	This study
NSPlD1 ΔvelB	NSlD	*niaD*$^{-}$ *sC*$^{-}$ *adeA*$^{-}$ *ΔargB::adeA*$^{-}$ *ΔligD::argB ΔpyrG::adeA AovelB:sC*	This study
Δ3 velB OE	ΔAoGprK-1ΔK-2ΔR	*niaD*$^{-}$ *sC*$^{-}$ *adeA*$^{-}$ *ΔargB::adeA*$^{-}$ *ΔligD::argB ΔpyrG::adeA ΔAogprK-1 ΔAogprK-2 ΔAogprR niaD*$^{-}$::(*PamyB-AovelB niaD)*	This study
Δ3 ΔvelB	ΔAoGprK-1ΔK-2ΔR	*niaD*$^{-}$ *sC*$^{-}$ *adeA*$^{-}$ *ΔargB::adeA*$^{-}$ *ΔligD::argB ΔpyrG::adeA ΔAogprK-1 ΔAogprK-2 ΔAogprR AovelB::sC*	This study

***S. cerevisiae* strain list**

**Strain**	**Host**	**Genotype**	**Reference**
BY4741		*MAT* a; *ura3* Δ0; *leu2* Δ0; *his3* Δ1; *met15* Δ0	Euroscarf (Frankfurt, Germany)
BY4741 (*URA3*$^{+}$, *HIS3*$^{+}$)	BY4741	*MAT* a; *ura3* Δ0; *leu2* Δ0; *his3* Δ1; *met15* Δ0; BYP5232 (P_{FUS1}-*lacZ URA3*); pYES2-His3 (*HIS3*)	This study
YO6055 (Δ*sst2*)	BY4741	*MAT* a; *ura3* Δ0; *leu2* Δ0; *his3* Δ1; *met15* Δ0; *YLR452c::kanMX4*	Euroscarf
YO6055 Control (Δ*sst2*, *URA3*$^{+}$, *HIS3*$^{+}$)	Y06055	*MAT* a; *ura3* Δ0; *leu2* Δ0; *his3* Δ1; *met15* Δ0; *YLR452c::kanMX4*; BYP5232 (P_{FUS1}-*lacZ URA3*); pYES2-His3 (*HIS3*)	This study
YO6055 K-1 RGS (AoGprK-1 RGS)	Y06055	*MAT* a; *ura3* Δ0; *leu2* Δ0; *his3* Δ1; *met15* Δ0; *YLR452c::kanMX4*; BYP5232 (P_{FUS1}-*lacZ URA3*); pYES2-His3 (P_{GAL1}-*AogprK-1 rgs HIS3*)	This study
YO6055 K-1 RGS (AoGprK-2 RGS)	Y06055	*MAT* a; *ura3* Δ0; *leu2* Δ0; *his3* Δ1; *met15* Δ0; *YLR452c::kanMX4*; BYP5232 (P_{FUS1}-*lacZ URA3*); pYES2-His3 (P_{GAL1}-*AogprK-2 rgs HIS3*)	This study
YO6055 R RGS (AoGprR RGS)	Y06055	*MAT* a; *ura3* Δ0; *leu2* Δ0; *his3* Δ1; *met15* Δ0; *YLR452c::kanMX4*; BYP5232 (P_{FUS1}-*lacZ URA3*); pYES2-His3 (P_{GAL1}-*AogprR rgs HIS3*)	This study

Table S3. Primer list

Primer name	Sequence (5' to 3')	Target gene	Comments
GW ΔgprK-1 Up-F	GGGGACAACTTTGTATAGAAAAGTTGAGATGTACGGAAGGATTCTATTAAT	*AogprK-1*	Construct AoGprK-1 deletion strain (Gateway cloning system)
GW ΔgprK-1 Up-R	GGGGACTGCTTTTTTGTACAAACTTGGATGGCGGTGTTTCGCTGAA	*AogprK-1*	Construct AoGprK-1 deletion strain (Gateway cloning system)
GW ΔgprK-1 DR-F	GGGGACAGCTTTCTTGTACAAAGTGGAAGGGTGGGCCTTTGTGACTTC	*AogprK-1*	Construct AoGprK-1 deletion strain (Gateway cloning system)
GW ΔgprK-1 DR-R	GATGGCGGTGTTTCGCTGAAAGAAT	*AogprK-1*	Construct AoGprK-1 deletion strain (Gateway cloning system)
GW ΔgprK-1 Down-F	ATTCTTTCAGCGAAACACCGCCATCTCGGCTTCTCGGGCTCGT	*AogprK-1*	Construct AoGprK-1 deletion strain (Gateway cloning system)
GW ΔgprK-1 Down-R	GGGGACAACTTTGTATAATAAAGTTGCCTCCAAACCCCTTCCCTACAA	*AogprK-1*	Construct AoGprK-1 deletion strain (Gateway cloning system)
GW ΔgprK-2 Up-F	GGGGACAACTTTGTATAGAAAAGTTGAGCCCACTCGCGGTCGCTGTCTATA	*AogprK-2*	Construct AoGprK-2 deletion strain (Gateway cloning system)
GW ΔgprK-2 Up-R	GGGGACTGCTTTTTTGTACAAACTTGGATGTGCGATGTTTCTCGTACTACA	*AogprK-2*	Construct AoGprK-2 deletion strain (Gateway cloning system)
GW ΔgprK-2 DR-F	GGGGACAGCTTTCTTGTACAAAGTGGGAAAACCGAACGGGACTATTTGC	*AogprK-2*	Construct AoGprK-2 deletion strain (Gateway cloning system)
GW ΔgprK-2 DR-R	GATGTGCGATGTTTCTCGTACTACA	*AogprK-2*	Construct AoGprK-2 deletion strain (Gateway cloning system)
GW ΔgprK-2 Down-F	TGTAGTACGAGAAACATCGCACATCCTAGCCTGTAGACAGCACGG	*AogprK-2*	Construct AoGprK-2 deletion strain (Gateway cloning system)
GW ΔgprK-2 Down-R	GGGGACAACTTTGTATAATAAAGTTGGATCCATGCGATTAGGTTATGCAGG	*AogprK-2*	Construct AoGprK-2 deletion strain (Gateway cloning system)
GW ΔgprR Up-F	GGGGACAACTTTGTATAGAAAAGTTGGCTTGTGAGCAATACGGAGTAACG	*AogprR*	Construct AoGprR deletion strain (Gateway cloning system)
GW ΔgprR Up-R	GGGGACTGCTTTTTTGTACAAACTTGACTTGATGGTCACTGGTCACGAA	*AogprR*	Construct AoGprR deletion strain (Gateway cloning system)
GW ΔgprR DR-F	GGGGACAGCTTTCTTGTACAAAGTGGCAGGTCAACCCATTGCACCAATT	*AogprR*	Construct AoGprR deletion strain (Gateway cloning system)
GW ΔgprR DR-R	ACTTGATGGTCACTGGTCACGAA	*AogprR*	Construct AoGprR deletion strain (Gateway cloning system)
GW ΔgprR Down-F	TCTTCGTGACCAGTGACCATCAAGTTTAAAGATAGAGAACTTCCGGGAG	*AogprR*	Construct AoGprR deletion strain (Gateway cloning system)
GW ΔgprR Down-R	GGGGACAACTTTGTATAATAAAGTTGACACCACGGGAGATTGGAAAC	*AogprR*	Construct AoGprR deletion strain (Gateway cloning system)
F-Native gprK-1	ACAACGAGCTGGGAACCCCACTGGTCTGTAGCCGAGAT	*AogprK-1*	Construct AoGprK-1 native promoter strain
R-gprK-1	CGCCCTTGCTCACCATCCCGACAGCCTCCTCCTTAATG	*AogprK-1*	Construct AoGprK-1 native promoter strain
F-Native gprK-2	ACAACGAGCTGGGAACCCCTCACTGGTGTAGATGGCTG	*AogprK-2*	Construct AoGprK-2 native promoter strain
R-gprK-2	GATTGACATGAAGACTGTCCCCATGGTGAGCAAGGGCG	*AogprK-2*	Construct AoGprK-2 native promoter strain
F-Native gprR	ACAACGAGCTGGGAACCCCAACACATCAAGCAAGGGTT	*AogprR*	Construct AoGprR native promoter strain
R-gprR	ACAACGCCCTTGCTCACCATCCCGAATGTGATGGACAAGTCGTC	*AogprR*	Construct AoGprR native promoter strain
F-Inver K-1 RGS 7TMx	ATGTGGGAGGTGATGCGCCA	AoGprK-1 RGS domain	Construct AoGprK-1 RGS domian strain
R-Inver K-1 RGS 7TMx	GATGGCGGTGTTTCGCTGAA	AoGprK-1 RGS domain	Construct AoGprK-1 RGS domian strain
F-Inver K-2 RGS 7TMx	ATGTGGGAGGTGAGGCGCCG	AoGprK-2 RGS domain	Construct AoGprK-2 RGS domian strain
R-Inver K-2 RGS 7TMx	GATGTGCGATGTTTCTCGTA	AoGprK-2 RGS domain	Construct AoGprK-2 RGS domian strain
F-Inver R RGS 7TMx	ATGTCCAGAAAGCAGCGAGA	AoGprR RGS domain	Construct AoGprR RGS domian strain
R-Inver R RGS 7TMx	ACTTGATGGTCACTGGTCAC	AoGprR RGS domain	Construct AoGprR RGS domian strain
R-AoGprK-1 7TM RGSx	ACAACGCCCTTGCTCACCATCCCGCAGGGAAGAAAGACAGTG	AoGprK-1 7TM region	Construct AoGprK-1 7TM region strain
R-AoGprK-2 7TM RGSx	ACAACGCCCTTGCTCACCATCCCGCAGGGAATAATGATGGTG	AoGprK-2 7TM region	Construct AoGprK-2 7TM region strain
R-AoGprR 7TM RGSx	ACAACGCCCTTGCTCACCATCCCGACAATAAGCTCATAACAT	AoGprR 7TM region	Construct AoGprR 7TM region strain
Inv-E87,N88AMutK1-F	GTCGCCTTCCTCACCAGCGTCGCCG	AoGprK-1 RGS domain	Construct AoGprK-1 RGS EN/AA point mutation strain
Inv-E87,N88AMutK1-R	AGCAGCGCCGGAAAAGTCGTTGAGGG	AoGprK-1 RGS domain	Construct AoGprK-1 RGS EN/AA point mutation strain
Inv-E87,N88AMutK2-F	ATTGCCTTCCTGGTGAGCGTGTCTC	AoGprK-2 RGS domain	Construct AoGprK-2 RGS EN/AA point mutation strain
Inv-E87,N88AMutK2-R	AGCAGCACCAGAGAAGTCATGAAGAG	AoGprK-2 RGS domain	Construct AoGprK-2 RGS EN/AA point mutation strain
Inv-E87,N88AMutR-F	ATCATCTTCCTGAATTATGTGCGAG	AoGprR RGS domain	Construct AoGprR RGS EN/AA point mutation strain
Inv-E87,N88AMutR-R	AGCAGCGCCGCTGAATTCCTTCACTG	AoGprR RGS domain	Construct AoGprR RGS EN/AA point mutation strain
L-6myc-gprK-1,2&R-RGS-NotI-F	ATTTGCGGCCGCATGGAGCAGAAACTGATTT	AoGprK-1,2 R RGS domain	Insertion of class VI GPCR RGS domein for pheromon assay
L-6myc-gprK1-RGS-XbaI-R	GCTCTAGATCAGACAGCCTCCTCCTTAA	AoGprK-1 RGS domain	Insertion of AoGprK-1 RGS domein for pheromon assay
L-6myc-gprK2-RGS-XbaI-R	GCTCTAGATTAGACAGTCTTCATGTCAATCCCA	AoGprK-2 RGS domain	Insertion of AoGprK-2 RGS domein for pheromon assay
L-6myc-gprR-RGS-XbaI-R	GCTCTAGACAGAATGTGATGGACAAGTCGT	AoGprR RGS domain	Insertion of AoGprR RGS domein for pheromon assay
pUtNAN-gpaA-F	GAATTCGAGCTCGGTACCCATGGGTTCGTGTGTAAGCA	*AogpaA*	Construct *AogpaA* in pUt-NAN plasmid
pUtNAN-gpaA-R	CCCTCTACTACAGATCCCCCTACAAGATACCTGAATCCTT	*AogpaA*	Construct *AogpaA* in pUt-NAN plasmid
pUtNAN-gpaB-F	GAATTCGAGCTCGGTACCCATGGGTTGTATGGGCTCAA	*AogpaB*	Construct *AogpaB* in pUt-NAN plasmid

pUtNAN-gpaB-R	CCCTCTACTACAGATCCCCTCATAAGATAAGAGTGTGCAG	*AogpaB*	Construct *AogpaB* in pUt-NAN plasmid
pUtNAN-ganA-F	GAATTCGAGCTCGGTACCCATGGGTTGCGGAATGAGTA	*AoganA*	Construct *AoganA* in pUt-NAN plasmid
pUtNAN-ganA-R	CCCTCTACTACAGATCCCCTTAAATCAGACCACAGAGTC	*AoganA*	Construct *AoganA* in pUt-NAN plasmid
AogpaA mutant F	GGACtcCGCAGCGAGCGGA	*AogpaA*	Insertion of mutation AG(3253 ~ 3254) → TC / Q → L in *AogpaA*
AogpaA mutant R	ACCCACATCAAACATGTGTATGCTCAGCTG	*AogpaA*	Insertion of mutation AG(3253 ~ 3255) → TC / Q → L in *AogpaA*
AogpaB mutant F	TGGCCtcCGGTCTGAGCGC	*AogpaB*	Insertion of mutation AA(3262 ~ 3263) → TC / Q → L in *AogpaB*
AogpaB mutant R	CCAACATCCATCATTCGGAAGTTCA	*AogpaB*	Insertion of mutation AA(3262 ~ 3264) → TC / Q → L in *AogpaB*
AoganA mutant F	GGTGGTCtcCGTTCCGAACG	*AoganA*	Insertion of mutation AA(3244 ~ 3245) → TC / Q → L in *AoganA*
AoganA mutant R	GACGTCGAACATCCGGTATGTC	*AoganA*	Insertion of mutation AA(3244 ~ 3246) → TC / Q → L in *AoganA*
F- gpaA 1000 up	AGCTCGGTACCCGGGGATCGCTGCTCCAGTCATGTAC	*AogpaA*	Construct *gpaA* deletion strain
R- gpaA 1000 up	CATCTGCGAGATCGGATCGGTTGGCGGGTATTTAGTCA	*AogpaA*	Construct *gpaA* deletion strain
F- gpaA 1000 down	AATCTCAGAAAATCCGCGGTGATTAGGGTGCTTTGATTG	*AogpaA*	Construct *gpaA* deletion strain
R- gpaA 1000 down	AGGTCGACTCTAGAGGATCGTTGAGGGCCTGGACC	*AogpaA*	Construct *gpaA* deletion strain
F- gpaB 1000 up	AGCTCGGTACCCGGGGATCCCGGTCCACGTTTTAC	*AogpaB*	Construct *gpaB* deletion strain
R- gpaB 1000 up	CATCTGCGAGATCGGATCGCTCGGATTATATTCTAGACAGC	*AogpaB*	Construct *gpaB* deletion strain
F- gpaB 1000 down	AATCTCAGAAAATCCGCGGACTTGAATGCTCTGCGC	*AogpaB*	Construct *gpaB* deletion strain
R- gpaB 1000 down	AGGTCGACTCTAGAGGATCCCTACTGTTGAGAGACTACC	*AogpaB*	Construct *gpaB* deletion strain
F- ganA 1000 up	AGCTCGGTACCCGGGGATCCGATGTACCACTTGTGG	*AoganA*	Construct *ganA* deletion strain
R- ganA 1000 up	CATCTGCGAGATCGGATCGGTTGTCTATGATTTTCTCTCAC	*AoganA*	Construct *ganA* deletion strain
F- ganA 1000 down	AATCTCAGAAAATCCGCGGCAGAGACGATTGGCTTCC	*AoganA*	Construct *ganA* deletion strain
R- gnaA 1000 down	AGGTCGACTCTAGAGGATCCGGTTAGAAGTCGTCTGAG	*AoganA*	Construct *ganA* deletion strain
F-velB OE	GAATTCGAGCTCGGTACCCATGTATGCTATCGAAGAAA	*velB*	Construct *velB* overexpress strain
R-velB OE	ACAAGAAAGCTGGGTCCCCATCGTAATCGTCCCCATCG	*velB*	Construct *velB* overexpress strain
F- velB 1000 up	AGCTCGGTACCCGGGGATCCTGTACGTGATTTTAAAGA	*velB*	Construct *velB* deletion strain
R- velB 1000 up	ACATCTGCGAGATCGGATCGGGCGTGAGAGTGGAATAAA	*velB*	Construct *velB* deletion strain
F- velB 1000 down	AAATCTCAGAAAATCCGCGGGAAAGTCGTGTTGCTTTGC	*velB*	Construct *velB* deletion strain
R- velB 1000 down	AGGTCGACTCTAGAGGATCTGGATACTCTCCTGCTCTT	*velB*	Construct *velB* deletion strain
F- sC	CGATCCGATCTCGCAGATG	*sC*	Construct *velB* deletion strain (*sC* marker)
R- sC	AATCTCAGAAAATCCGCGG	*sC*	Construct *velB* deletion strain (*sC* marker)
qPCR velB-F	TCAACGTGGGTACTCAATCCAG	*velB*	Investigation of gene expression level using qRT-PCR
qPCR velB-R	GGGAAATTTCTTGGCGGAGAAG	*velB*	Investigation of gene expression level using qRT-PCR
qPCR laeA-F	TTCTTGGGACCTGATTCACCTG	*laeA*	Investigation of gene expression level using qRT-PCR
qPCR laeA-R	TCGATCTCCACCTGTTCAAACC	*laeA*	Investigation of gene expression level using qRT-PCR
qPCR veA-F	ACTTCCACCTGTACGAGGAAAC	*veA*	Investigation of gene expression level using qRT-PCR
qPCR veA-R	TCGAGACGAAACTCCAGGAATG	*veA*	Investigation of gene expression level using qRT-PCR
qPCR vosA-F	CGTCAATCGACTTTGGTAACCG	*vosA*	Investigation of gene expression level using qRT-PCR
qPCR vosA-R	ACCCTTGCATGATAGGAGTTGG	*vosA*	Investigation of gene expression level using qRT-PCR
qPCR nsdD F2	TGTGCCCAATGAGCCTATGG	*nsdD*	Investigation of gene expression level using qRT-PCR
qPCR nsdD R2	CCGAAGTGATAGAACCGGCA	*nsdD*	Investigation of gene expression level using qRT-PCR
qPCR sclR F2	TAGGGACCATCTGCCAGCTC	*sclR*	Investigation of gene expression level using qRT-PCR
qPCR sclR R2	ACGACGTTGGGTATGAGCAC	*sclR*	Investigation of gene expression level using qRT-PCR

*Underlined letters in the sequence indicate the sequence of the plasmid

*Small letters in the sequence indicate the mutant sequence point

```
AoGpaA            MGCGMS---- --TEDK---- ----EGKARN EEIENQLKRD KMMQRNEIKM LLLGAGESGK STILKQMKLI
AoGpaB            MGSCVSTEPA DN-------- ----EPKKRS QAIDRRLEED SRRLRRECKI LLLGSGESGK STIVKQMKII
AoGanA            MGCMGS---- --KPVDTTDK DALQRNARID KVLKN----D KKVMDRTIKI LLLGAGESGK STIIKQMRII
Gpa1              MGCTVS---- --TQTIGDES DPFLQNKRAN DVIEQSLQLE KQRDKNEIKL LLLGAGESGK STVLKQLKLL
Gi                MGCTLS---- ---------- AEDKAAVERS KMIDRNLRED GEKAAREVKL LLLGAGESGK STIVKQMKII
Gs                MGCLGN---- ---SKTEDQR NEEKAQREAN KKIEKQLQKD KQVYRATHRL LLLGAGESGK STIVKQMRIL
Clustal Consensus**.  .                              . . : . .     :        :: ****:***** **::**::::

AoGpaA            HEGGYSRD-- ---------- ---ERESFKE IIYSNTVQSM RVILEAMESL ELPLEDAR-- ----------
AoGpaB            HQNGYTVE-- ---------- ---ELALYRL TVCKNLLDCA KSLVGAYHQF SLEPSSQK-- ----------
AoGanA            HSGGFPDD-- ---------- ---ERRQTRA VIYSNVVIAF KVLLDIMRTE SIEFEQEK-- ----------
Gpa1              HQGGFSHQ-- ---------- ---ERLQYAQ VIWADAIQSM KILIIQARKL GIQLDCDDPI NNKDLFACKR
Gi                HEAGYSEE-- ---------- ---ECKQYKA VVYSNTIQSI IAIIRAMGRL KIDFGDSAR- ----------
Gs                HVNGFNGEGG EEDPQAARSN SDGEKATKVQ DIKNNLKEAI ETIVAAMSNL VPPVELANP- ----------
Clustal Consensus*   *:   :              *       :   :    .    ::

AoGpaA            HEYHVQTIFM QPAQIEGDNL PP-------- ---------- ---------- ---------- ----------
AoGpaB            VRDYVQYISD YNIDPDPHTT LD-------- ---------- ---------- ---------- ----------
AoGanA            TKPLADYMDT LESDVGSDEA FS-------- ---------- ---------- ---------- ----------
Gpa1              ILLKAKALDY INASVAGGSD FLNDYVLKYS ERYETRRRVQ STGRAKAAFD EDGNISNVKS DTDRDAETVT
Gi                -ADDARQLFV LAGAAEEGFM TA-------- ---------- ---------- ---------- ----------
Gs                -ENQFRVDYI LSVMNVPDFD FP-------- ---------- ---------- ---------- ----------
Clustal Consensus

AoGpaA            ---------- ---------- ---------- ---------- ---------- EVGNAIGALW R-DSGVQECF
AoGpaB            ---------- ---------- ---------- ---------- ---------A KVGEAITYIW N-DPCTSTVL
AoGanA            ---------- ---------- ---------- ---------- --------DL KVRDAMRDMW N-DAGVQKAV
Gpa1              QNEDADRNNS SRINLQDICK DLNQEGDDQM FVRKTSREIQ GQNRRNLIHE DIAKAIKQLW NNDKGIKQCF
Gi                ---------- ---------- ---------- ---------- ---------- ELAGVIKRLW K-DSGVQACF
Gs                ---------- ---------- ---------- ---------- ---------P EFYEHAKALW E-DEGVRACY
Clustal Consensus                                                       . .     :*  .  *

AoGpaA            KRSREYQLND SAKYYFDAIE RIAQPDYLPT DQDVLRSRVK TTGITETTFI IGDLTYRMFD VGGQRSERKK
AoGpaB            EHQNEFYLMD SAPYFFEEAK RIASPDFIPN VNDVLRARTK TTGIYETRFT MGQLSIHMFD VGGQRSERKK
AoGanA            ARGHEFALHD NLHYFFDSLD RIFAPGWLPD NQDMLQARLR TTGITETLFE LGGQMNFRMMD VGGQRSERKK
Gpa1              ARSNEFQLEG SAAYYFDNIE KFASPNYVCT DEDILKGRIK TTGITETEFN IGSSKFKVLD AGGQRSERKK
Gi                NRSREYQLND SAAYYLNDLD RIAQPNYIPT QQDVLRTRVK TTGIVETHFT FKDLHFKMFD VGGQRSERKK
Gs                ERSNEYQLID CAQYFLDKID VIKQADYVPS DQDLLRCRVL TSGIFETKFQ VDKVNFHMFD VGGQRDERRK
Clustal Consensus :  .*:  *  .     *:::   .   :    . .::   :*:*:  *   *:**  **  *   .  .  :::* .****.**:*

AoGpaA            WIHCFENVTT ILFLVAISEY DQLLFEDETV NRMQEALTLF DSICNSRWFV KTSIILFLNK IDRFKEKLPV
AoGpaB            WIHCFENVTS IIFCVALSEY DQVLLEESNQ NRMMESLVLF DSVVNSRWFM RTSIILFLNK VDLFRQKLPR
AoGanA            WIHCFEGVQC LLFMVALSGY DQCLVEDQNA NQMHEAMMLF ESLVNGEWFK RKPIILFLNK IDLFKGKLSV
Gpa1              WIHCFEGITA VLFVLAMSEY DQMLFEDERV NRMHESIMLF DTLLNSKWFK DTPFILFLNK IDLFEEKVKS
Gi                WIHCFEGVTA IIFCVALSDY DLVLAEDEEM NRMHESMKLF DSICNNKWFT DTSIILFLNK KDLFEEKIKK
Gs                WIQCFNDVTA IIFVVASSSY NMVIREDNQT NRLQEALNLF KSIWNNRWLR TISVILFLNK QDLLAEKVLA
Clustal Consensus**:**:. :    ::*  :*  *  *   :   :  *:.   *::  *:: **  .:: *. .*:     ..******  *  :   *:

AoGpaA            --SPMKNYFP DYEGG-ADYA AACDYILN-- ---------- ---RFVSLNQ AEQKQIYTHF TCATDTTQIR
AoGpaB            --SPLSNYFP DYSGG-NDVN RAAKYLLW-- ---------- ---RFNQVNR AH-LNLYPHL TQATDTTNIR
AoGanA            --SPVSKHFP DYNGSNTDFD AAARYFAD-- ---------- ---RFRGINR IPDREIYIHY TNATDTTLLK
Gpa1              --MPIRKYFP DYQGRVGDAE AGLKYFEK-- ---------- ---IFLSLNK TN-KPIYVKR TCATDTQTMK
Gi                --SPLTICYP EYAGSNTYEE AAAYIQCQ-- ---------- ---FEDLNKR KDTKEIYTHF TCATDTKNVQ
Gs                GKSKIEDYFP EFARYTTPED ATPEPGEDPR VTRAKYFIRD EFLRISTASG DGRHYCYPHF TCAVDTENIR
Clustal Consensus         :    :*  ::                                   .        *  :   *  * .**   ::

AoGpaA            FVMAAVNDII IQENLRLCGL I
AoGpaB            LVFAAVKETI LQNALKDSGI L
AoGanA            ATMDSVQDMI IQKNLHTLIL -
Gpa1              FVLSAVTDLI IQQNLKKIGI I
Gi                FVFDAVTDVI IKNNLKDCGL F
Gs                RVFNDCRDII QRMHLRQYEL L
Clustal Consensus .:      :  *    :    *:     :
```

Fig. S1

Sequence alignment of three Gα proteins in *A. oryzae*, AoGpaA, AoGpaB, and AoGanA, with yeast Gpa1, human Gαi, and human Gαs

Box in purple, the N-terminal myristoylation and palmitoylation sequences. Box in red, the guanine nucleotide binding motifs (GXGXXGKS). Box in blue, the GTPase domains (DXXGQ). To exogenously express GTPase-deficient mutants, the constructs in which Gln residue in the GTPase domain marked by the red asterisk was mutated to Leu were introduced to the control strain.

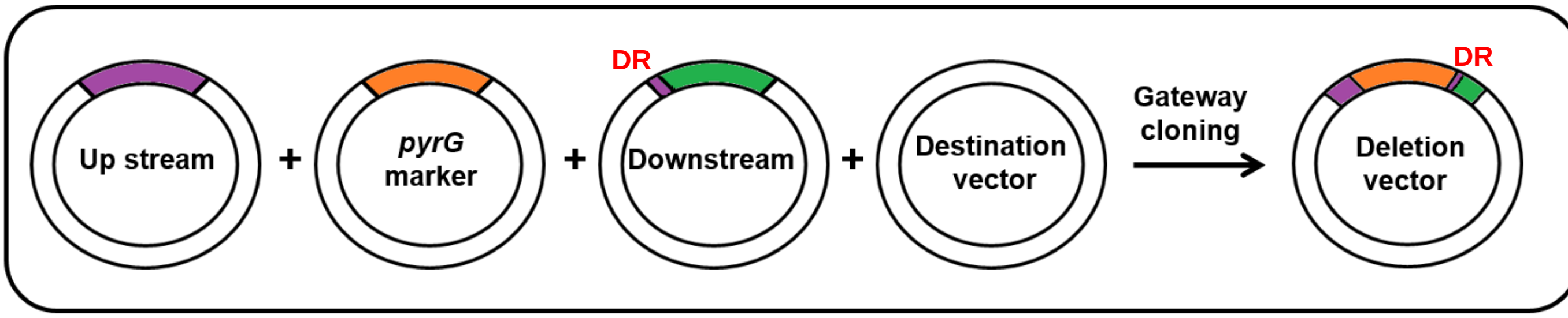

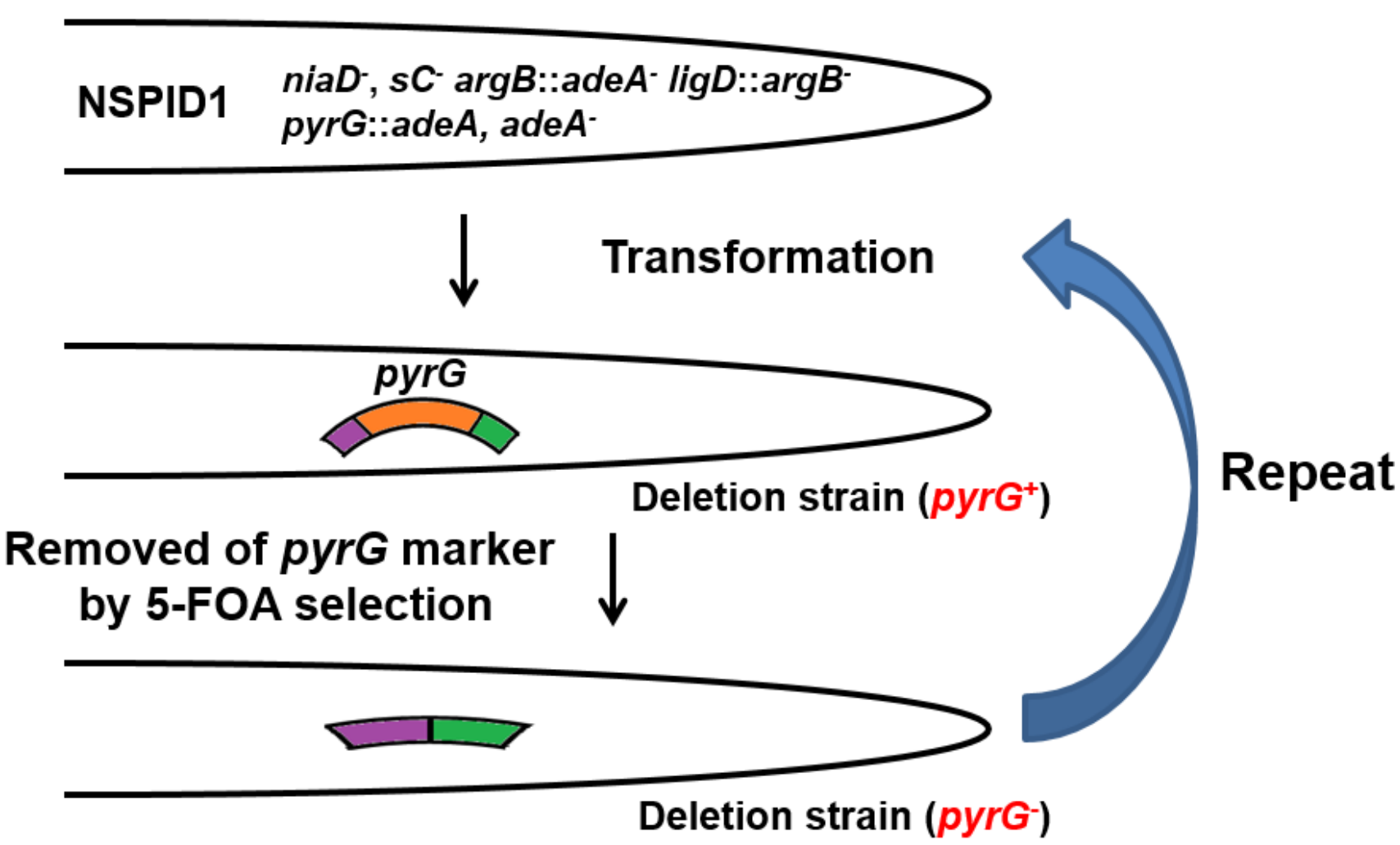

Host strain	Constructed strain
NSPID1	Each single deletion strains
ΔAogprK-1	ΔAogprK-1<br>ΔAogprK-2
ΔAogprK-1	ΔAogprK-1<br>ΔAogprR
ΔAogprR	ΔAogprK-2<br>ΔAogprR
ΔAogprK-1ΔK-2	ΔAogprK-1<br>ΔAogprK-2<br>ΔAogprR

Figure S2

Construction of single and multiple gene deletion mutants using the marker recycling system

Strains with single and multiple gene deletion were constructed using the *pyrG* marker recycling system as described in the Materials and methods. The *pyrG*$^+$ transformants with the deletion of target gene were grown on the medium containing 5-FOA to eliminate *pyrG* marker by homologous recombination. The resultant *pyrG*$^-$ hosts were used for the subsequent gene deletion. The host and constructed strains are shown in the table in the right.

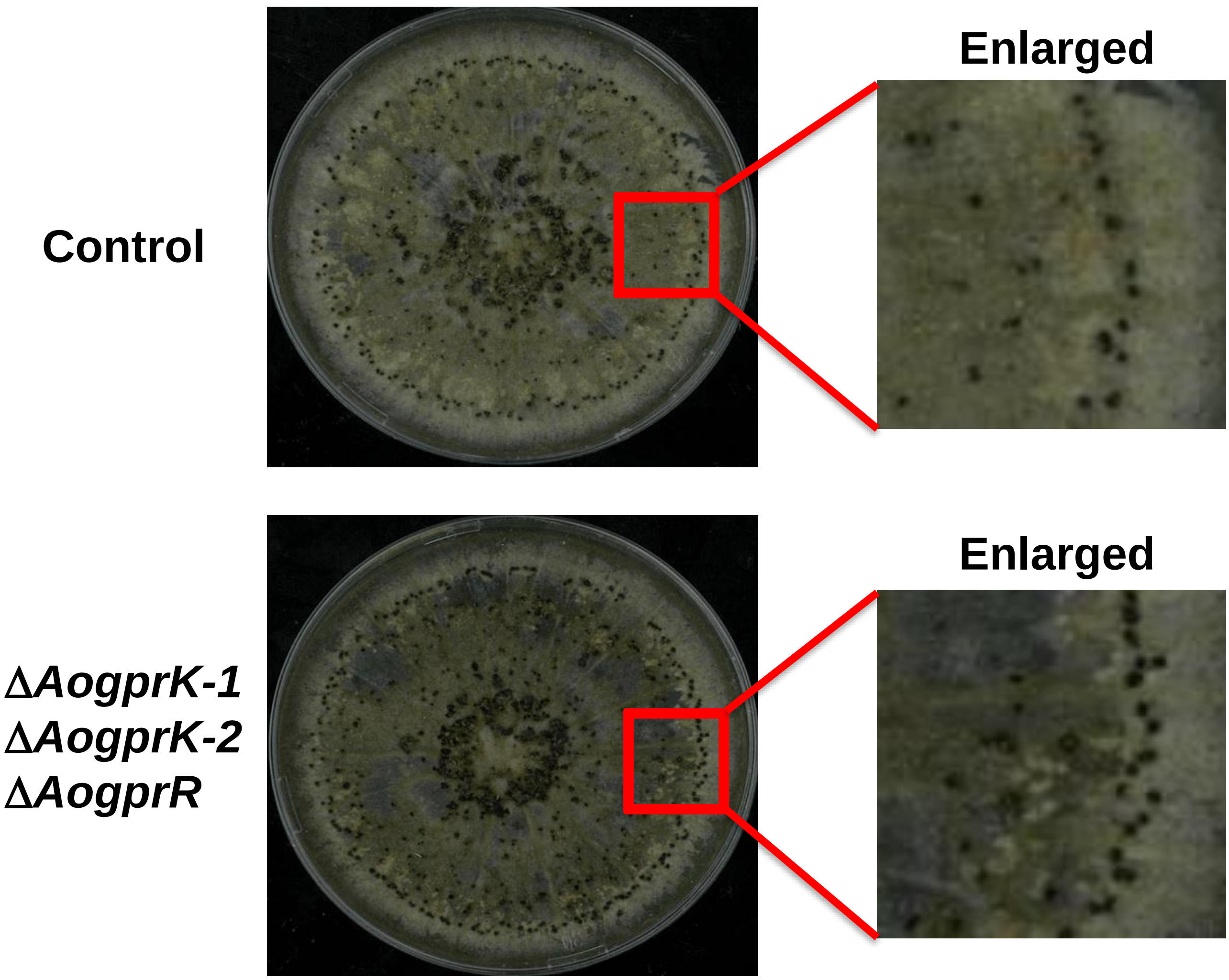

Fig. S3

Images of plates of the control and the triple gene deletion mutant

Control and the triple gene deletion mutant were grown on the DPY agar plates containing uridine and uracil for 20 days at 30°C. The plates were washed with 70% ethanol and observed from the bottom.

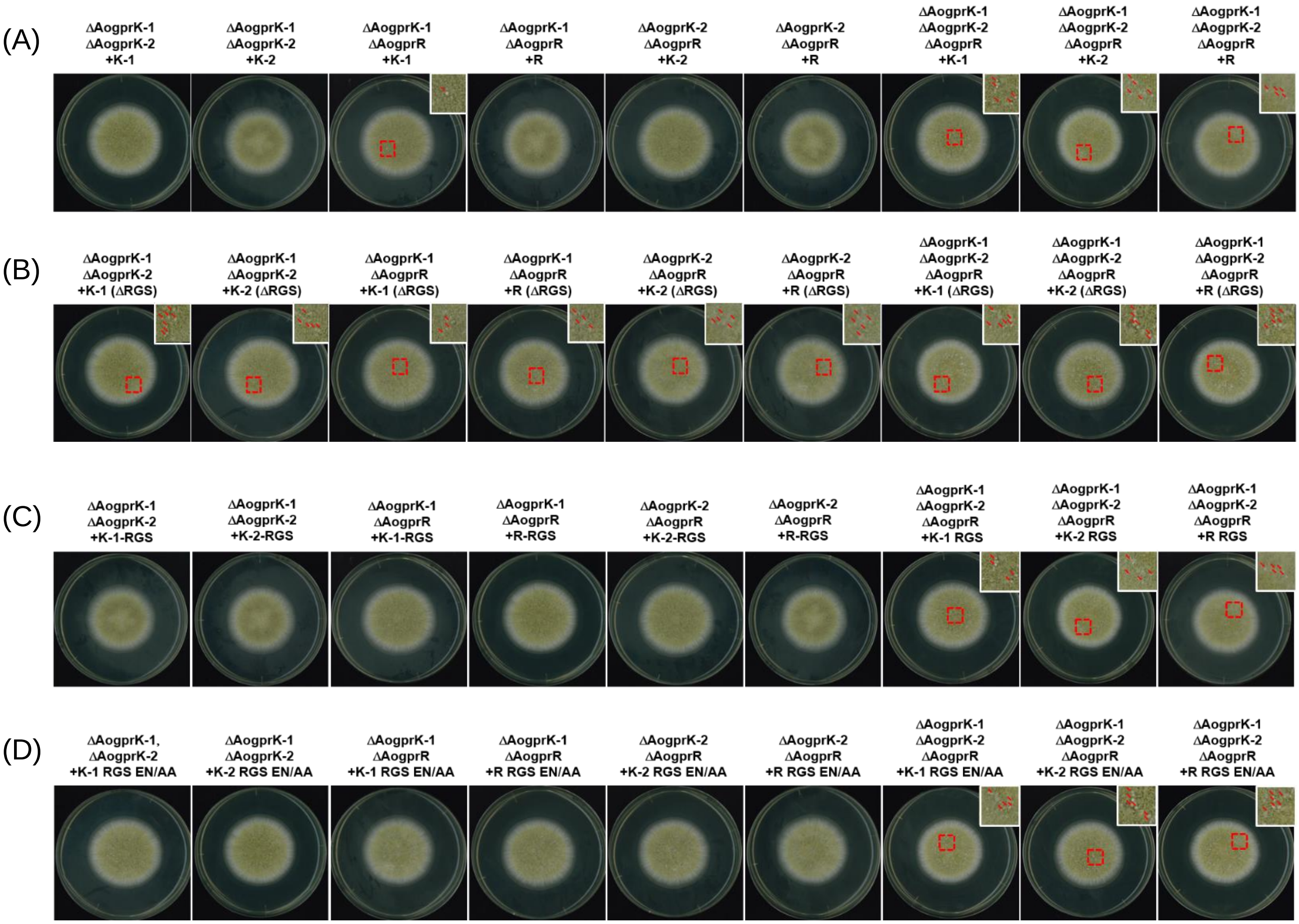

Fig. S4

Sclerotia formation in the complemented strains

Conidial suspensions (10^3/5 μL) were spotted on the DPY agar plates containing uridine and uracil, and incubated for 4 days at 30°C. Sclerotia formation in the strains complemented by the full-length constructs (A), the constructs containing only the 7-TM domain (B), the constructs containing only the RGS domain (C), and the full-length constructs containing EN/AA mutation in the RGS domains (D), respectively, was observed. The genotypes of the host strains are shown at the top, and the complemented gene is indicated by '+' (e.g. '+AogprK-1' indicates that the AogprK-1 was complemented, while 'ΔRGS' indicates that only the 7-TM domain was complemented).

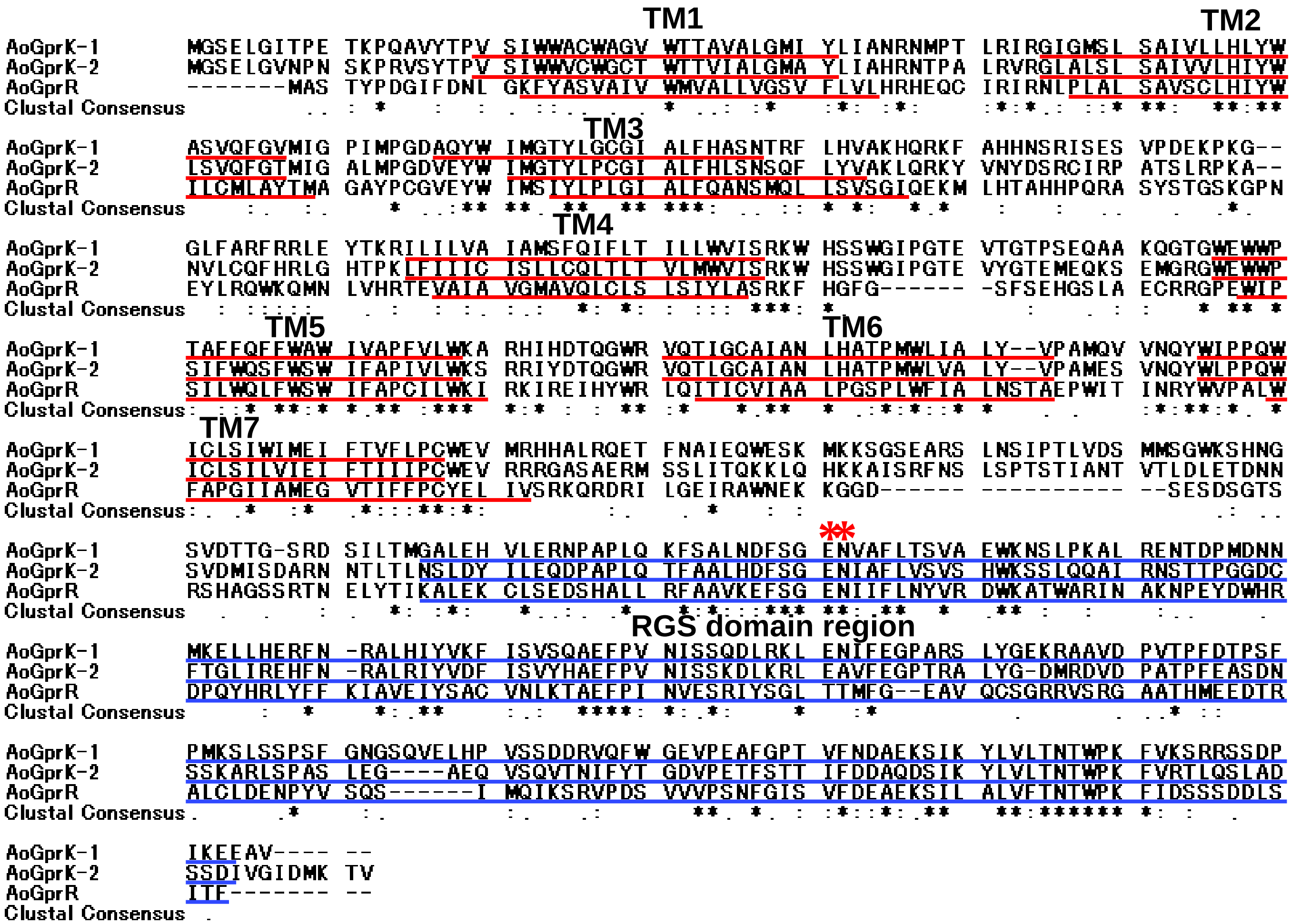

Fig. S5

Sequence alignment of *A. oryzae* class VI GPCRs

The amino acid sequences of *A. oryzae* class VI GPCRs are aligned using Clustal W (https://www.genome.jp/tools-bin/clustalw). The underlines in red and blue indicate the transmembrane and RGS domains, respectively. For the complementation analysis, residues 1-360 in AoGprK-1, 1-361 in AoGprK-2, and 1-333 in AoGprR were used as the 7-TM domains; residues 361-559 in AoGprK-1, 362-561 in AoGprK-2, and 334-522 in AoGprR were used as the RGS domains. Amino acid residues (EN) shared by human RGS4 and mutated to AA in this study are marked by red asterisks.

(A)

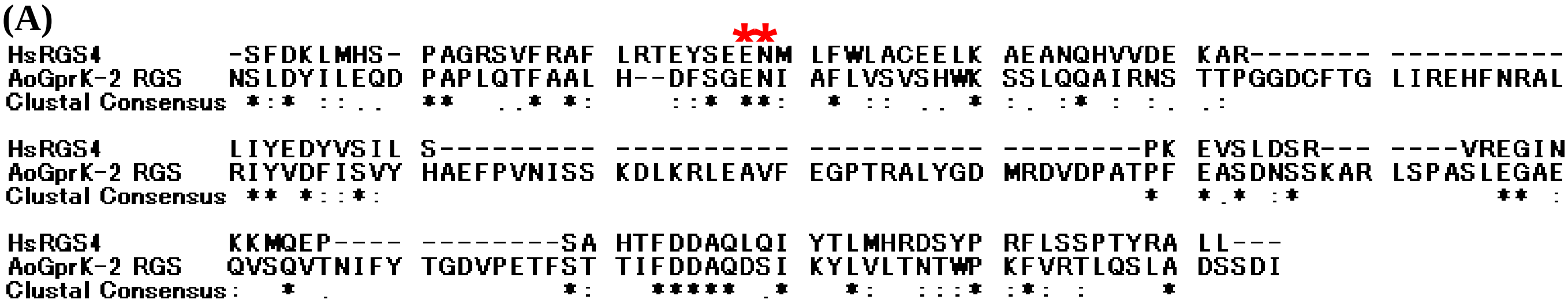

(B)

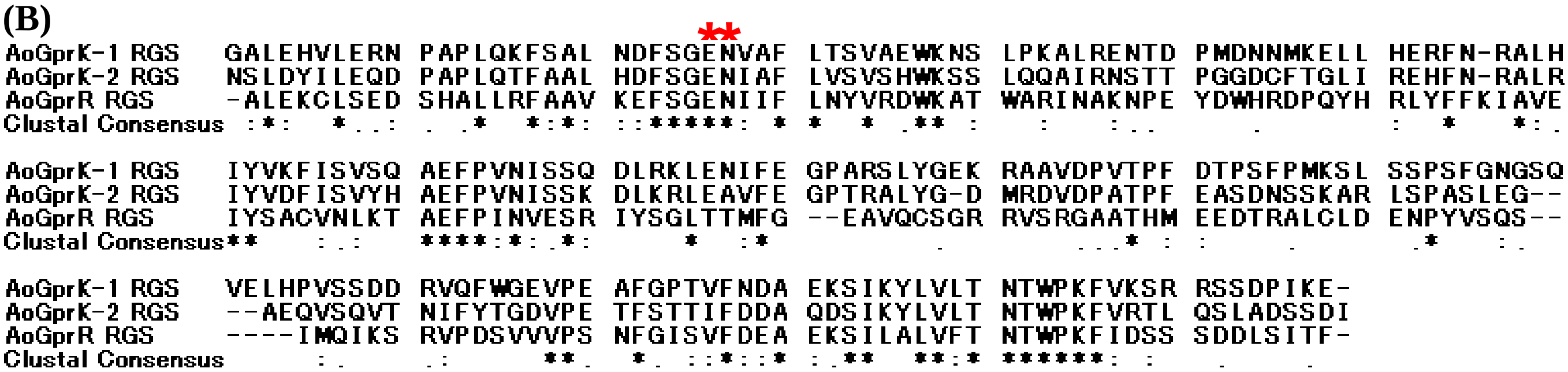

Fig. S6

Sequence alignment of RGS domains of class VI GPCRs and human RGS4

(A) Sequence alignment of RGS domain of AoGprK-2 and human RGS4 (accession: P49798). Conserved EN residues are marked by red asterisks. (B) Sequence alignment of RGS domains of AoGprK-1, AoGprK-2, and AoGprR. Conserved EN residues that were mutated to AA in this study are marked by red asterisks.

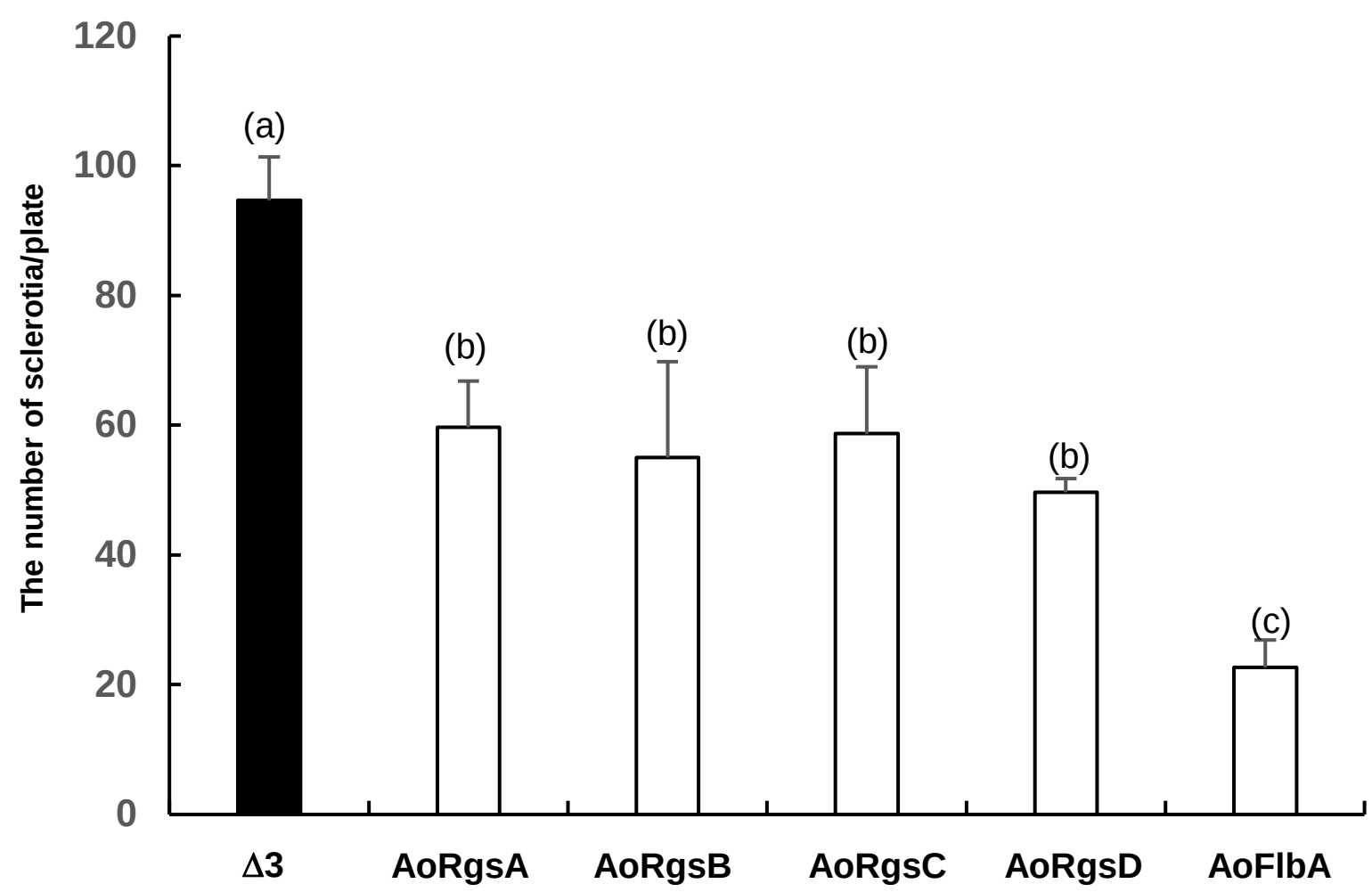

Fig. S7

Sclerotia formation in the strains overexpressing RGS domain-containing proteins in the triple gene deletion background (Δ3)

The triple gene deletion mutant (Δ3) and the strains overexpressing RGS domain-containing proteins (RgsA, RgsB, RgsC, RgsD, and FlbA) in the Δ3 background were grown on DPY+UU agar plate for 4 d at 30°C and the sclerotia formation was examined. Different letters indicate that they show significant difference (n=3).

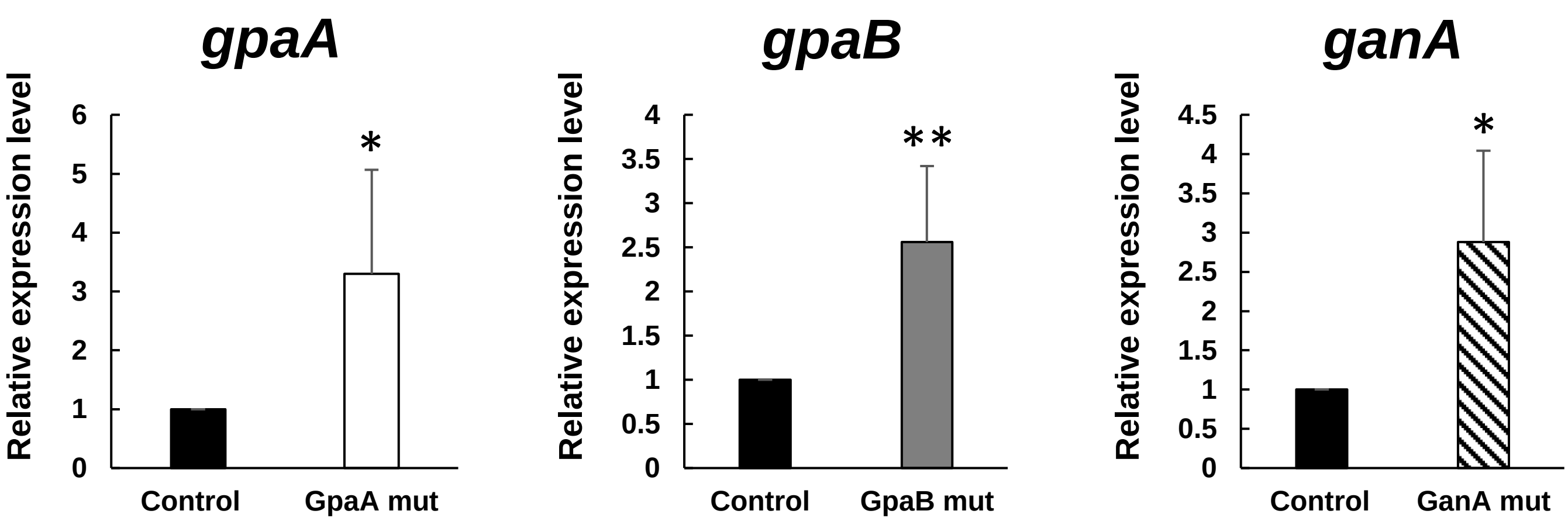

Fig. S8

Relative expression levels of the genes encoding Gα subunits in the control and the mutants overexpressing GTPase-deficient form of Gα (Gα mut strains)

Control and Gα mut strains were grown for 6 d at 30°C on the CD+Met agar plates containing dextrin instead of glucose as a carbon source. After incubation, the relative expression levels of each Gα were analyzed by qPCR. Error bars represent S.D. (* and **, $p < 0.05$ and $p < 0.01$ by Student's *t*-test, respectively; n=4).

(a)

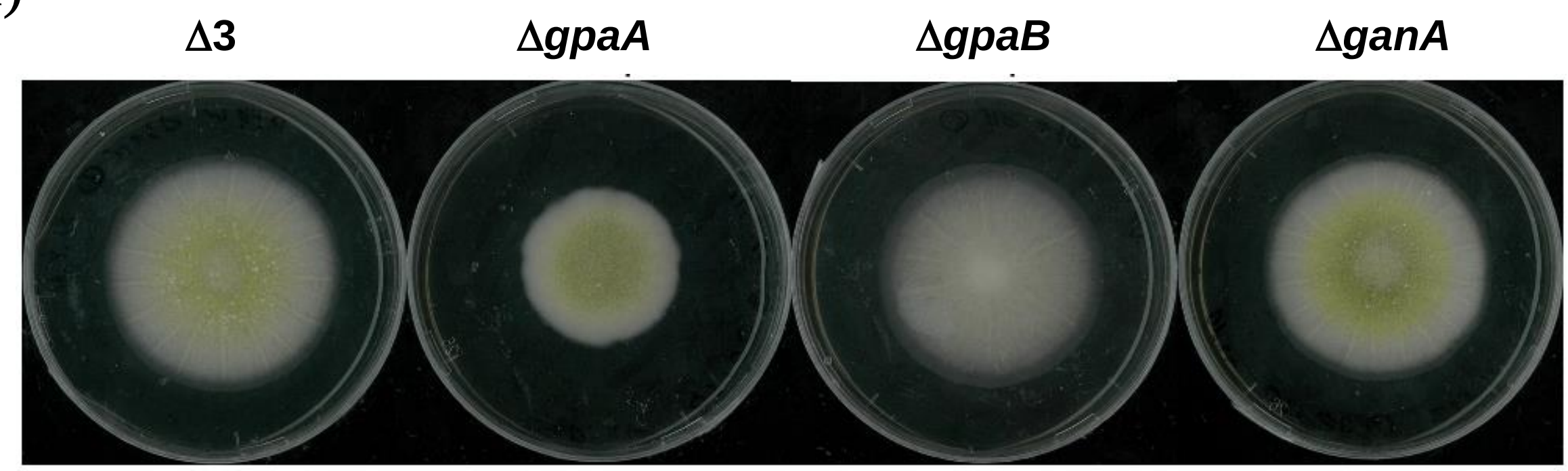

(b)

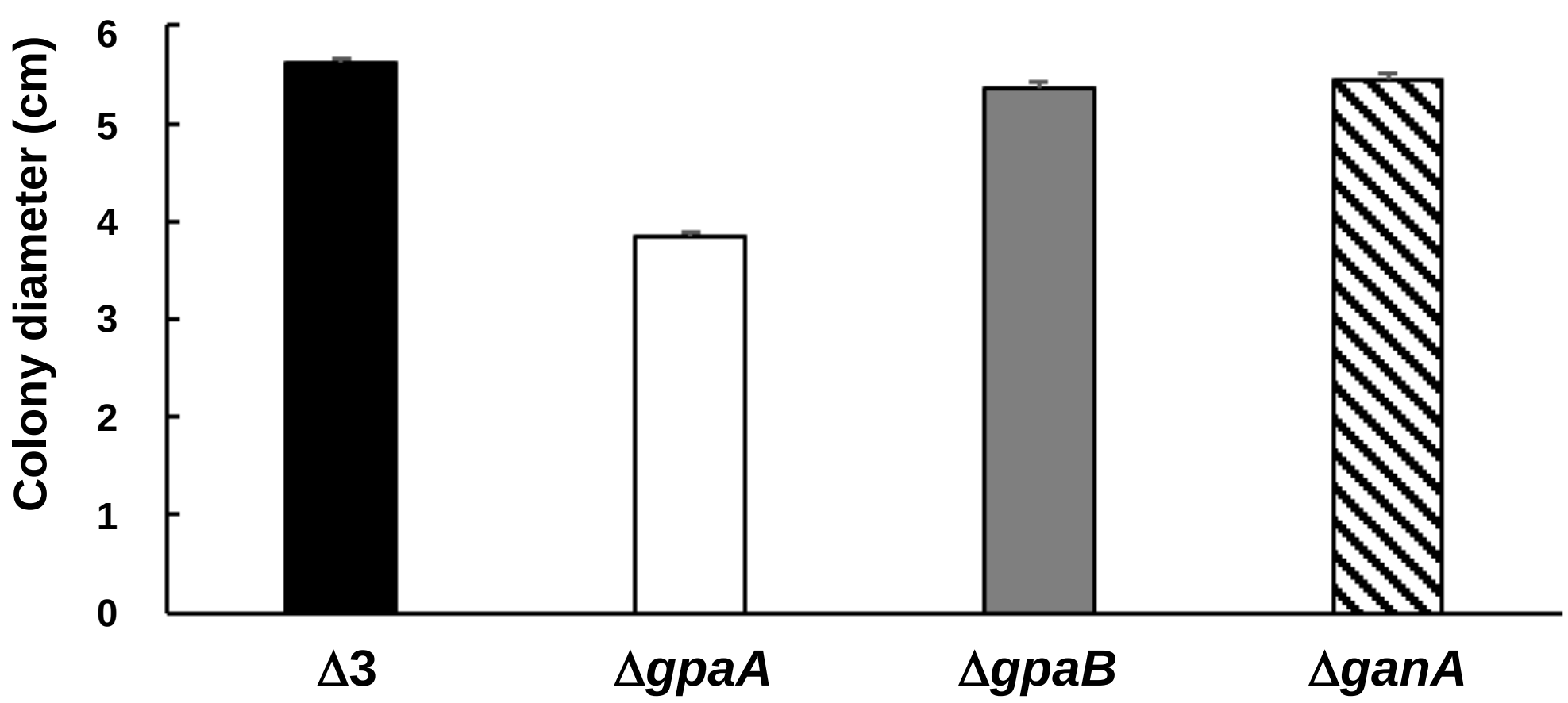

Fig. S9

Growth of the strains deleted for the genes encoding Gα subunits in the triple gene deletion background

The triple gene deletion mutant (Δ3) and the strains deleted for the genes encoding Gα subunits in the Δ3 background were grown on DPY+UU agar plate for 4 d at 30°C. Colony photograph (a) and colony diameter (b) (n=3) are shown.

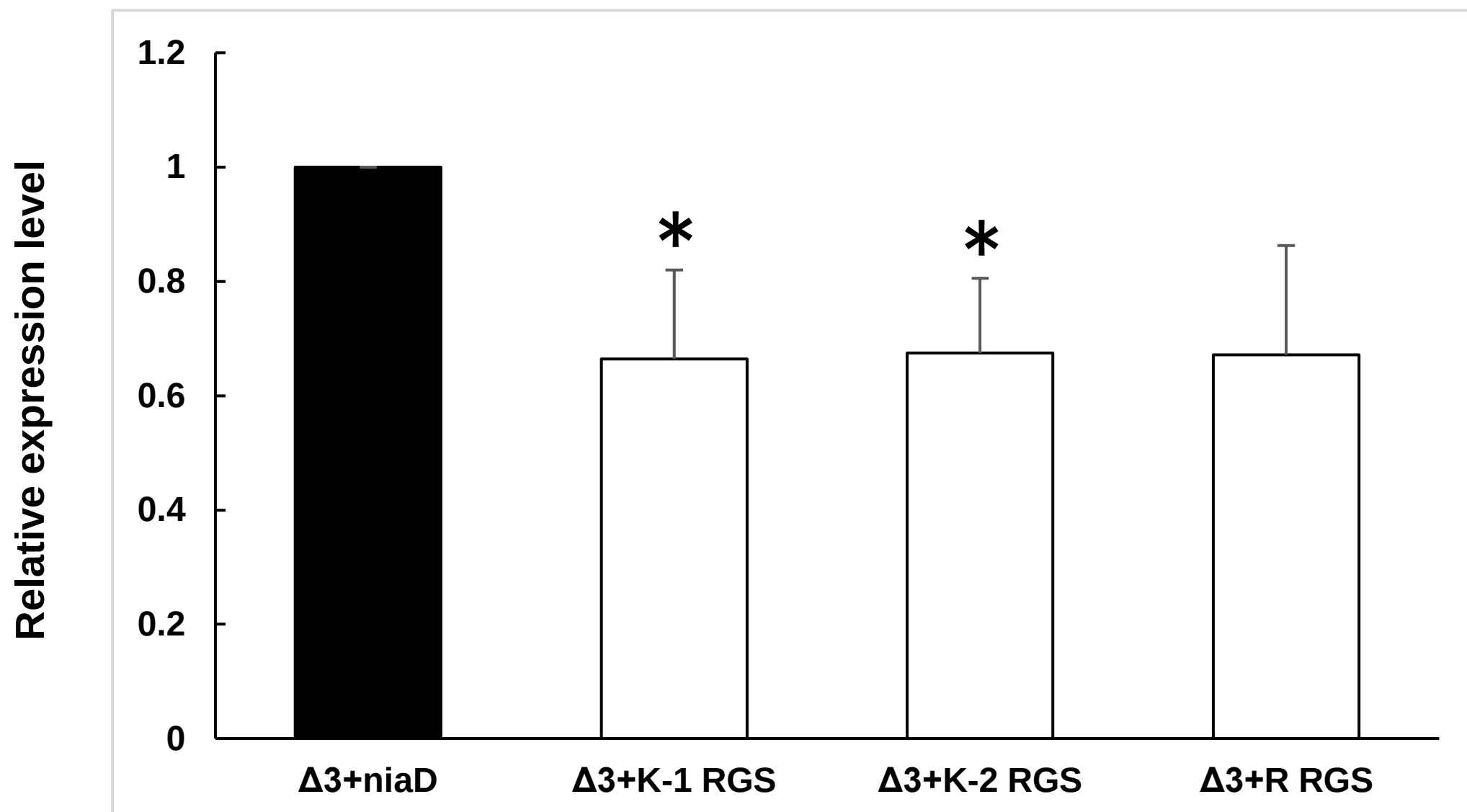

Fig. S10

Expression of *velB* in the triple gene deletion mutant complemented by one of the constructs carrying only the RGS domain of either AoGprK-1, AoGprK-2, or AoGprR

The triple gene deletion mutant (Δ3) transformed by the vector (Δ3+niaD) or one of the constructs carrying only the RGS domain of either AoGprK-1, AoGprK-2, or AoGprR were examined for the expression of *velB*. Error bars represent S.D. (*, $p < 0.05$ by Student's *t*-test; n=3).
